# Multistep energy and electron transfer processes in novel rotaxane donor–acceptor hybrids generating microsecond-lived charge separated states†
†Electronic supplementary information (ESI) available. See DOI: 10.1039/c5sc02895g



**DOI:** 10.1039/c5sc02895g

**Published:** 2015-10-02

**Authors:** Sabrina V. Kirner, Christian Henkel, Dirk M. Guldi, Jackson D. Megiatto Jr, David I. Schuster

**Affiliations:** a Department of Chemistry and Pharmacy and Interdisciplinary Center for Molecular Materials , Friedrich-Alexander-Universität Erlangen-Nürnberg , D-91058 Erlangen , Germany . Email: dirk.guldi@fau.de; b Department of Chemistry , New York University , New York , NY 10003 , USA . Email: david.schuster@nyu.edu

## Abstract

A new set of [Cu(phen)_2_]^+^ based rotaxanes, featuring [60]-fullerene as an electron acceptor and a variety of electron donating moieties, namely zinc porphyrin (ZnP), zinc phthalocyanine (ZnPc) and ferrocene (Fc), has been synthesized and fully characterized with respect to electrochemical and photophysical properties. The assembly of the rotaxanes has been achieved using a slight variation of our previously reported synthetic strategy that combines the Cu(i)-catalyzed azide–alkyne cycloaddition reaction (the “click” or CuAAC reaction) with Sauvage's metal-template protocol. To underline our results, complementary model rotaxanes and catenanes have been prepared using the same strategy and their electrochemistry and photo-induced processes have been investigated. Insights into excited state interactions have been afforded from steady state and time resolved emission spectroscopy as well as transient absorption spectroscopy. It has been found that photo-excitation of the present rotaxanes triggers a cascade of multi-step energy and electron transfer events that ultimately leads to remarkably long-lived charge separated states featuring one-electron reduced C_60_ radical anion (C_60_˙^–^) and either one-electron oxidized porphyrin (ZnP˙^+^) or one-electron oxidized ferrocene (Fc˙^+^) with lifetimes up to 61 microseconds. In addition, shorter-lived charge separated states involving one-electron oxidized copper complexes ([Cu(phen)_2_]^2+^ (*τ* < 100 ns)), one-electron oxidized zinc phthalocyanine (ZnPc˙^+^; *τ* = 380–560 ns), or ZnP˙^+^ (*τ* = 2.3–8.4 μs), and C_60_˙^–^ have been identified as intermediates during the sequence. Detailed energy diagrams illustrate the sequence and rate constants of the photophysical events occurring with the mechanically-linked chromophores. This work pioneers the exploration of mechanically-linked systems as platforms to position three distinct chromophores, which are able to absorb light over a very wide range of the visible region, triggering a cascade of short-range energy and electron transfer processes to afford long-lived charge separated states.

## Abstract 2

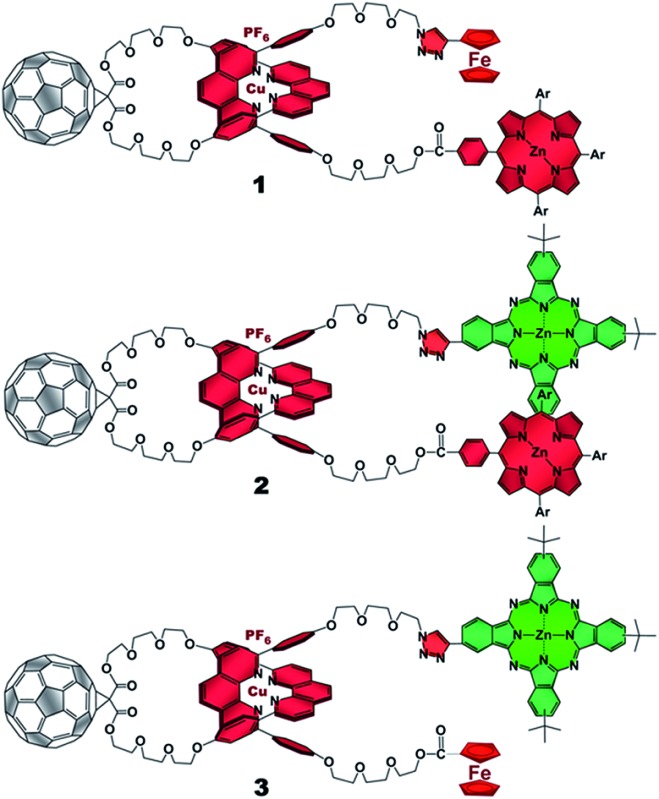
A new set of [Cu(phen)_2_]^+^ based rotaxanes, featuring [60]-fullerene as an electron acceptor and a variety of electron donating moieties, namely zinc porphyrin (ZnP), zinc phthalocyanine (ZnPc) and ferrocene (Fc), has been synthesized and fully characterized.

## Introduction

In more than 25 years of fullerene research, C_60_ emerged as an excellent electron acceptor in electron transfer reactions, due to its unique properties. For instance, the rigid C_60_ cage can accept up to six electrons and has a very small reorganization energy in electron transfer reactions. Consequently, C_60_ has been utilized in photosynthetic reaction mimics, photovoltaics, and catalysis.^1^ In order to guarantee efficient electron transfer, suitable electron donating moieties have to be chosen. In terms of photosynthetic reaction mimics, they have to fulfill the redox requirements imposed by the fullerene as well as to have the ability to efficiently harvest light. Both of these specifications are met by tetrapyrrolic macrocycles, such as porphyrins and phthalocyanines.

An interesting alternative to spatially arrange the chromophores is provided by mechanically-interlocked systems, such as catenanes and rotaxanes, decorated with electron donors and electron acceptors.^2–4^ These photo-active interlocked platforms have allowed the systematic investigation of the effects of molecular topology on the thermodynamic and kinetic parameters of the electron and energy transfer processes in artificial photosynthetic models. This effort is motivated by the need to better understand the intricate roles that the protein environment plays in the photo-induced processes in natural photosynthesis.^5–10^

For example, we have shown^11^ that catenanes decorated with zinc porphyrin (ZnP) and C_60_ ultimately yield a charge separated state possessing ZnP˙^+^ and C_60_˙^–^ radical ions with a lifetime roughly two times longer than the same charge separated state in the corresponding rotaxane.^12^ This significant difference in lifetime reflects the distinct topology of the two interlocked systems. The catenane is conformationally rigid, while the rotaxane counterpart is not. Therefore, the former keeps the ZnP and C_60_ moieties at longer and fixed distances, while the latter brings them closer to each other, a process that is driven by secondary interactions between the chromophores and allowed by the unclosed ring of the rotaxane.^11–19^

One of the well-established synthetic protocols to assemble rotaxanes and catenanes is Sauvage's Cu(i)-templated synthesis.^20–24^ In this synthetic strategy, two 1,10-phenanthroline moieties (phen) become orthogonally arranged as a result of their tetrahedral coordination to the Cu(i) template ion, which creates the cross-over points needed for the formation of the mechanical bond. In the case of artificial photosynthetic models, the Cu(i) template synthesis ensures a further benefit, namely the presence of an additional photoactive unit, the [Cu(phen)_2_]^+^ complex. It has been shown by us^12,25^ and others^26,27^ that the resulting [Cu(phen)_2_]^+^ complex facilitates the electronic communication between appended electron donors and electron acceptors on rotaxanes and catenanes upon photoexcitation.

**Fig. 1:**
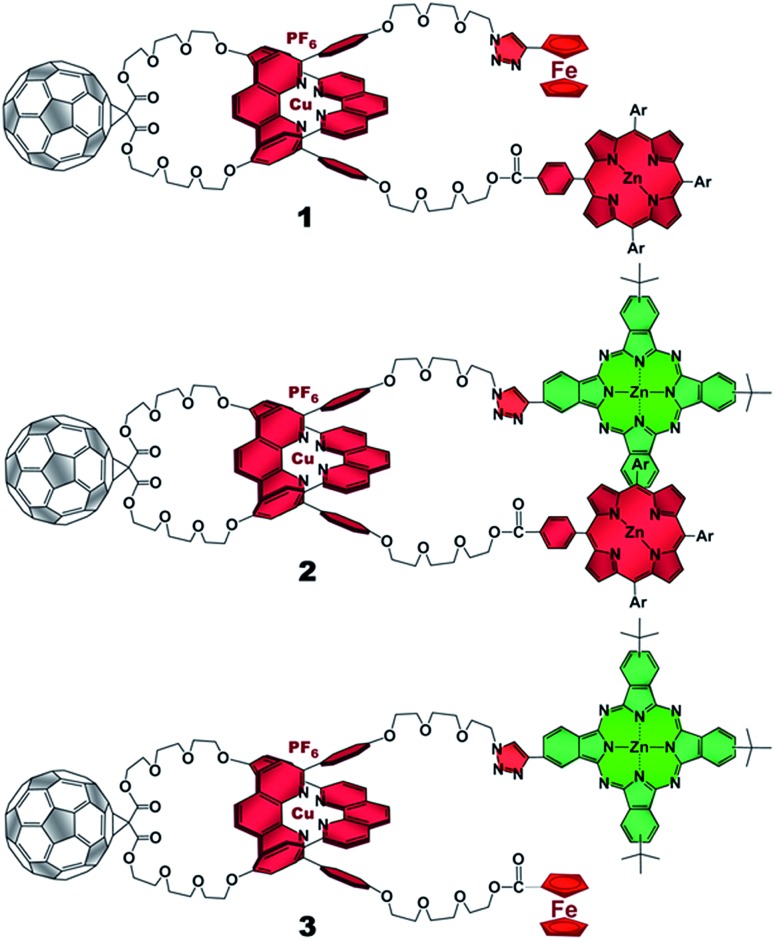
[Cu(phen)_2_]^+^–C_60_ based rotaxanes (**1–3**) stoppered by three different combinations of electron donors investigated in the present work. Ar = 3,5-di-*tert*-butylphenyl.

**Fig. 2:**
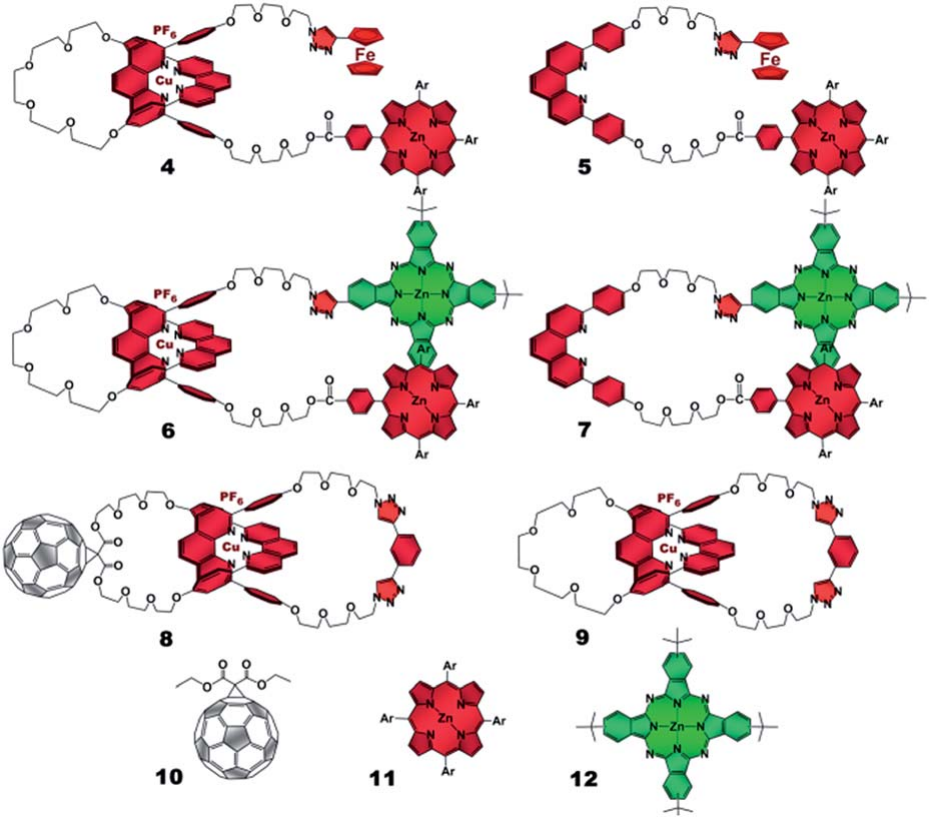
Rotaxane and catenane models as well as reference compounds used to investigate the photophysical processes of the target rotaxanes.

The present work reports that a minor variation of this strategy has allowed the incorporation of porphyrin, phthalocyanine, and ferrocene electron donors as stoppering groups of [Cu(phen)_2_]^+^–C_60_ based rotaxanes to afford a new family of multi-chromophoric interlocked structures (Fig. 1) that are able to undergo a cascade of energy and electron transfer reactions to yield charge separated states with remarkably long lifetimes. Furthermore, a complete set of model rotaxanes and catenanes lacking the electron donors, the C_60_ or both, were prepared using our synthetic protocol and probed as reference systems (Fig. 2). A complete and systematic investigation of the new interlocked compounds by electrochemistry, UV-Vis absorption, steady state and time resolved emission spectroscopies as well as transient absorption spectroscopy in the femtosecond and nanosecond time regime has allowed us to gather the rates of the energy and electron transfer, charge shift, and charge recombination processes occurring upon photo-excitation of the three [Cu(phen)_2_]^+^–C_60_ rotaxanes shown in Fig. 1 to elucidate their deactivation processes.

## Results and discussion

### 1. Synthesis

The synthetic strategy used to prepare rotaxanes **1–3** is depicted in Scheme S1 (see ESI†). Briefly, one of the hydroxyl groups of compound **13** ^11^ is tosylated under classical conditions to yield **14**, which undergoes a nucleophilic substitution with sodium azide to afford **15**. The remaining hydroxyl group in **15** is esterified using 1-ethyl-3-(3-dimethyl-aminopropyl)carbodiimide (EDC) as coupling agent and dimethylaminopyridine (DMAP) as base following the conditions reported in the experimental section (see ESI†) with either porphyrin **16** ^28^ or commercially available ferrocenecarboxylic acid **21** to produce the phen derivatives **17** and **22**, respectively. Threading of **17** or **22** through the C_60_-based phen-macrocycle **18** using [Cu(CH_3_CN)_4_]PF_6_ as the Cu(i) source in a mixture of dichloromethane/acetonitrile (7 : 3, v/v) at room temperature and under nitrogen atmosphere quantitatively yielded pseudo-rotaxanes **19** and **23**, respectively, as revealed by TLC. Finally, pseudo-rotaxanes **19** or **23** and commercially available alkynyl ferrocene or phthalocyanine **20** ^29^ were submitted to our “click” protocol^11,17,30^ to afford the target rotaxanes **1**, **2**, and **3**. Rotaxane and catenane model compounds were prepared following the same strategy from the appropriate building blocks.

### 2. Ground state interactions – absorption spectra and electrochemistry

**Fig. 3:**
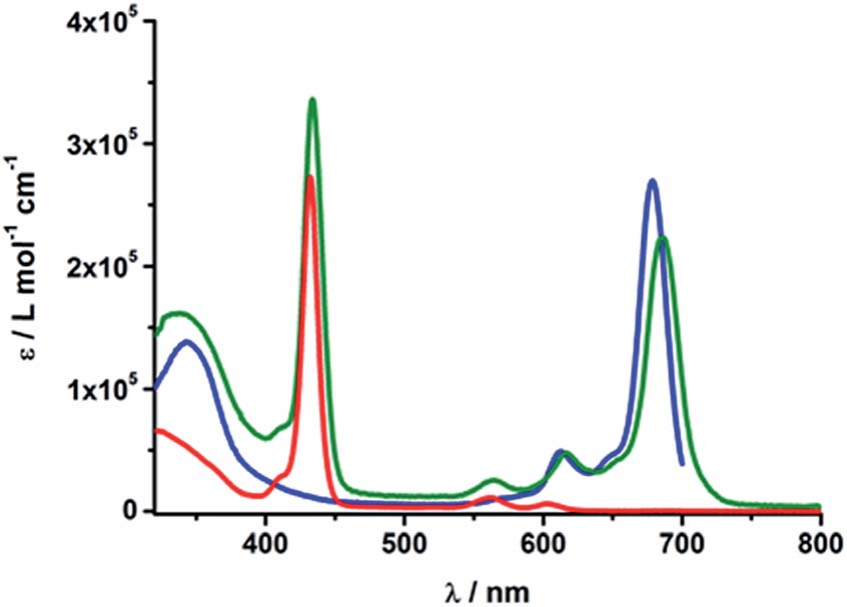
UV/Vis absorption spectra of Fc–ZnP–[Cu(phen)_2_]^+^–C_60_ **1** (red), ZnP–ZnPc–[Cu(phen)_2_]^+^–C_60_ **2** (olive) and Fc–ZnPc–[Cu(phen)_2_]^+^–C_60_ **3** (blue) in PhCN.

The absorption spectra of rotaxanes **1–3** feature the typical absorption characteristics of the various building blocks (Fig. 3). In particular, rotaxane **1** exhibits broad absorption between 300 and 380 nm, corresponding to C_60_ and [Cu(phen)_2_]^+^. ZnP with its Soret and Q-bands reveals absorptions at 432, 561, and 602 nm. Compared to ZnTPP **11**, the ZnP absorption bands exhibit a red shift of around 3 nm. Ferrocene is spectroscopically invisible in the 300 to 800 nm range. Rotaxane **2** features additional absorption maxima corresponding to ZnPc, namely Soret and Q bands at 340, 616, and 685 nm. Here, the ZnP absorption bands are 2 nm red shifted compared to **1** or masked by the ZnPc absorption. Similarly, the absorption of C_60_ and [Cu(phen)_2_]^+^ is covered by that of ZnPc. The absorption spectrum of rotaxane **3** is dominated by the features corresponding to ZnPc, that is, maxima at 345, 613 and 679 nm, which are ∼6 nm red shifted compared to ZnPc reference compound **12**. Increased extinction coefficients in the Soret band region stem from C_60_ and [Cu(phen)_2_]^+^ absorptions.

Turning to electrochemistry (see Table S1†) the redox chemistry of rotaxane **1** is best described as the superposition of catenane **8**, ZnTPP **11**, and ferrocene. In particular, two one-electron reductions are seen within the electrochemical window of the solvent (dichloro-methane) at –1.09 and –1.45 V (all redox potentials are relative to Fc/Fc^+^). Both are assigned to C_60_ centered reductions. Three oxidations are observed. As in rotaxane **4**, the first oxidation at +0.04 V is assigned to Fc, while the second, more intense oxidation at +0.24 V corresponds to a [Cu(phen)_2_]^+^-centered process as well as to the first ZnP oxidation. Likewise, the third oxidation at + 0.88 V correlates with the second ZnP oxidation.

Rotaxane **2** exhibits all the redox features seen for ZnTPP **11**, Zn^*t*^Bu_4_Pc **12**, and catenane **8**. To be more precise, two reductions at –1.13 and –1.54 V correspond to the reduction of C_60_, while the oxidation of [Cu(phen)_2_]^+^ is observed at +0.13 V. As seen for **6** and **7**, the first ZnP and ZnPc oxidations cannot be clearly distinguished. A broader and more intense feature at +0.18 V is attributed to both. The second ZnP oxidation is observed at +0.83 V.

Finally, rotaxane **3** features two oxidations as well as two reductions within the electrochemical window. Here, the ferrocene oxidation is slightly shifted to less negative potentials, namely –0.05 V, possibly due to interactions with the other chromophores, while the second oxidation with about twice the intensity, is assigned to the one-electron oxidations of ZnPc and [Cu(phen)_2_]^+^. The reductive scan indicates two reductions of C_60_ at –1.10 and –1.55 V. The redox potentials of compounds **1–12** are summarized in Table S1 in the ESI.†

### 3. Excited state interactions - fluorescence

Table S2† lists the fluorescence properties of the investigated rotaxanes as well as the corresponding reference compounds. The strongest fluorescent chromophore is ZnPc reference compound **12**, with a fluorescence quantum yield of 0.3.^31,32^ Its fluorescence spectrum shows a maximum at 779 nm in THF – Fig. S4† – and at 790 nm in PhCN – Fig. S5.† ZnTPP **11**, with maxima at 602 and 655 nm in THF and 609 and 660 nm in PhCN, exhibits much weaker fluorescence with a quantum yield of 0.04.

In contrast, the ZnP fluorescence in rotaxanes **1** and **4** is significantly quenched. Upon excitation at 420 nm, which coincides with the Soret band absorption maximum, a relatively weak ZnP fluorescence of 0.02 (Table S2†) is observed for **1** and **4**, while in **5** the ZnP fluorescence is not quenched at all, which suggests energy and/or electron transfer from ZnP to [Cu(phen)_2_]^+^ and C_60_ occurs in **1** and **4**.

**Fig. 4:**
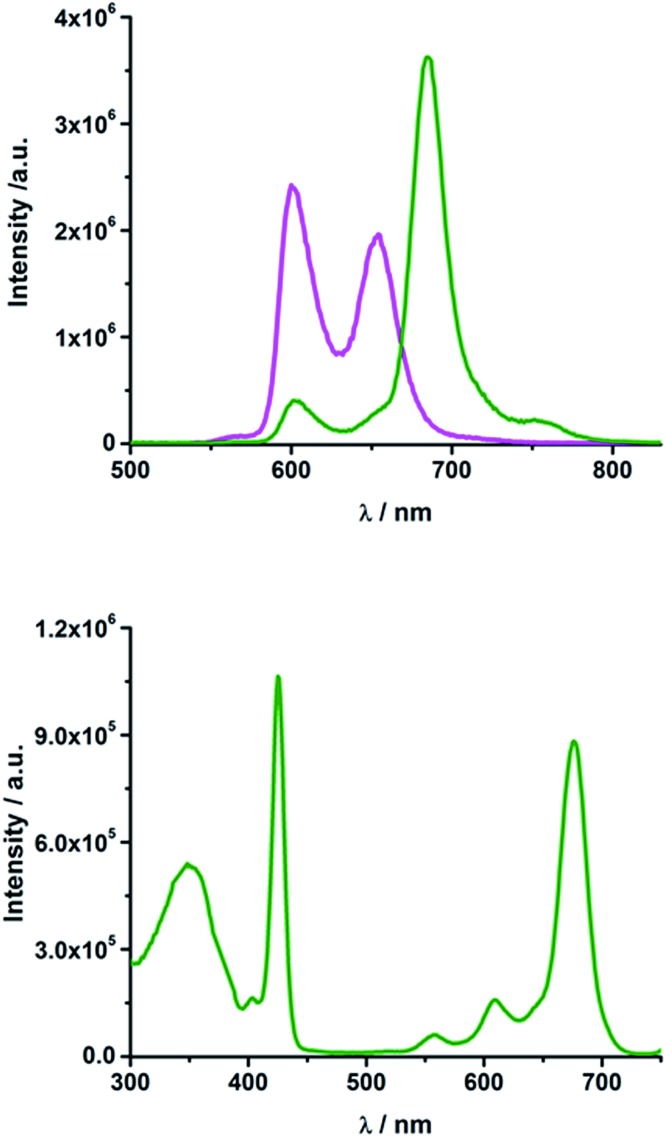
Top: Emission spectrum of rotaxane **2** (olive) and ZnTPP **11** (magenta) in THF upon excitation at 420 nm (OD = 0.084); bottom: excitation spectrum of rotaxane **2** in THF for emission at 760 nm.

In rotaxane **2**, as well as in reference compounds **6** and **7**, the ZnPc fluorescence is quenched compared to **12** and the maximum emission is shifted to longer wavelengths. They show ZnPc fluorescence quantum yields between 0.07 and 0.13 in THF (Table S2†) and between 0.09 and 0.24 in PhCN. The ZnP emission in rotaxanes **2** and **6** as well as in reference compound **7** is even more strongly quenched. Upon excitation into the porphyrin's Soret band at 420 nm only weak porphyrin fluorescence ranging from 0.004 to 0.008 (Table S2†) is observed, which is partly overlaid by a strong fluorescence maximizing at ∼690 nm with a shoulder at 760 nm (Fig. 4, top).

Glancing at the excitation spectrum of **2** (Fig. 4 bottom) it is clear that this fluorescence originates from ZnPc, since the excitation spectrum corresponding to the 760 nm emission combines features of ZnP as well as of ZnPc. In contrast, the excitation spectrum of the 600 nm feature clearly proves that this fluorescence originates exclusively from ZnP. Thus, we postulate that an energy transfer from ZnP (2.06 eV) to the energetically lower lying ZnPc (1.8 eV) takes place. The quantum yield for this energy transfer was found to be 0.04 for rotaxane **2** in THF.

Rotaxane **3** exhibits 50% quenched ZnPc fluorescence – Table S2.† This quenching gives rise to the conclusion that energy and/or electron transfer from ZnPc to [Cu(phen)_2_]^+^ and to C_60_ takes place in these rotaxanes.

### 4. Excited state interactions – transient absorption spectroscopy

To obtain information about the formation and decay processes of the excited states upon photoexcitation, transient absorption spectroscopy was carried out with rotaxanes **1–3** as well as reference compounds **4–12** (see ESI†) in THF using fs (387, 420 and 660 nm) as well as ns (355, 425 and 670 nm) laser excitation. Notably, we assume that the electron transfer processes are driven by through-space rather than through-bond interactions.

**Fig. 5:**
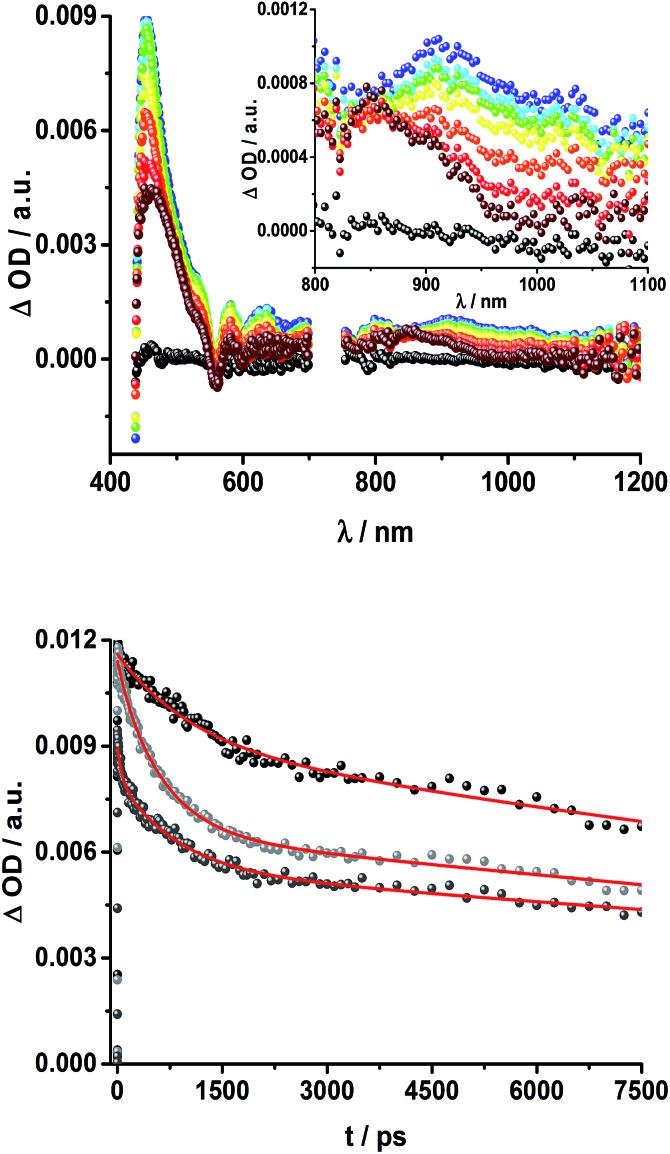
Top: Transient absorption spectrum (visible and near-infrared) registered upon femtosecond flash photolysis (420 nm, 150 nJ) of Fc–ZnP–[Cu(phen)_2_]^+^–C_60_ rotaxane **1** in THF with time delays between 0 (black) and 7.5 ns (wine) at room temperature. Inset: zoom into the near infrared region. Bottom: Time absorption profiles of **1** (dark grey), **4** (grey), and **5** (black) at 455 nm upon excitation with a 420 nm laser pulse, monitoring the decay of the ZnP singlet excited state.

The transient absorption spectrum of rotaxane **1** – Fig. 5 – is dominated by features that can be assigned to the singlet and triplet excited states ZnP˙^+^. In the near infrared region features corresponding to ^3^MLCT* and ^1^*C_60_ are discernible. As time progresses, all of the aforementioned characteristics are replaced by a weak maximum at 1020 nm, which correlates with the fingerprint absorption of the one electron reduced form of C_60_.^11,12,16,34^ This is stable on the 7.5 ns timescale of our experimental setup. The signature of the one electron oxidized form of either [Cu(phen)_2_]^+^, ZnP, or Fc – as a complement to the one electron reduced form of C_60_ – are not discernible due to dominating ZnP singlet and triplet absorption features. The fact that the broad near-infrared transient is formed independently of the excitation wavelength leads us to conclude that energy transfer from ZnP to [Cu(phen)_2_]^+^ takes place. When comparing rotaxanes **1** and **4**, the ZnP fluorescence is quenched by 50% relative to ZnTPP **11** in both cases. Thus, the lack of energy transfer to C_60_ is hypothesized (Fig. 5).

#### 4.1 Rotaxane **1**

**Fig. 6:**
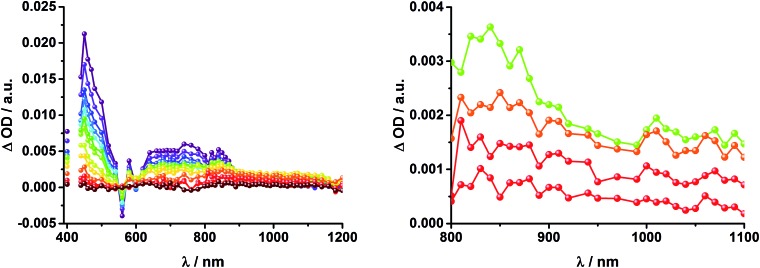
Left: Differential absorption spectra (visible and near infrared) registered upon nanosecond flash photolysis (425 nm, 5 mJ) of Fc–ZnP–[Cu(phen)_2_]^+^–C_60_ rotaxane **1** under aerobic conditions in THF with time delays between 200 ns (purple) and 15 μs (wine) at room temperature. Right: Differential absorption spectra (near infrared) registered upon nanosecond flash photolysis (425 nm, 5 mJ) of Fc–ZnP–[Cu(phen)_2_]^+^–C_60_ rotaxane **1** under aerobic conditions in THF with time delays between 1 (green) and 5 μs (red) at room temperature.

**Fig. 7:**
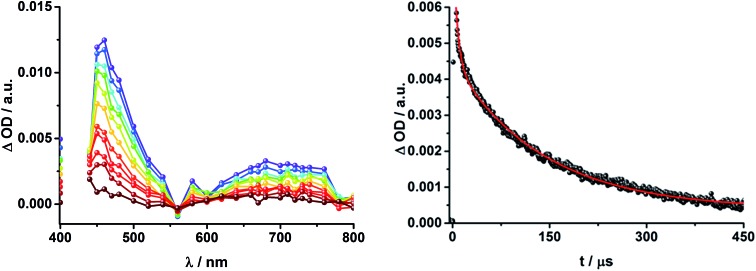
Left: Differential absorption spectra (visible) registered upon nanosecond flash photolysis (425 nm, 5 mJ) of Fc–ZnP–[Cu(phen)_2_]^+^–C_60_ rotaxane **1** under argon atmosphere in THF with time delays between 8 μs (purple) and 400 μs (wine) at room temperature. Right: Time absorption profile of the spectra on the left at 460 nm, monitoring the decay of the ZnP triplet excited state.

Complementary transient absorption measurements on the ns timescale under aerobic conditions as well as in argon-saturated THF shed light upon the excited state interactions in rotaxane **1**. Upon excitation at 425 nm, the differential absorption spectra are dominated by ZnP-centered features, namely the ZnP triplet excited state with a broad absorption throughout the visible region and maxima at 460 and 840 nm (Fig. 6). In the absence of oxygen, a ZnP triplet excited state lifetime of 142 ± 12 μs was determined for rotaxane **1** in THF (Fig. 7). Notable is the fact that the triplet excited state of C_60_ gives rise to a transient maximum at 740 nm, whose formation is masked by the dominating ZnP triplet excited state features.^11,12^ A closer look at the differential absorption changes reveals additional peaks discernible at 680 and 1010 nm (Fig. 6). In line with previous reports, the latter is assigned to the one electron reduced form of C_60_, while the former correlates with the one electron oxidized form of ZnP.^11,14,15,18,33,35^ From the corresponding extinction coefficients, that is, 0.82 × 10^4^ M^–1^ cm^–1^ for the ZnP triplet excited state at 840 nm ^36^ and 1.5 × 10^4^ M^–1^ cm^–1^ for the one electron reduced form of C_60_,^37^ we conclude that the major product is the former. However, exact values for the yields of charge separation could not be determined. Accordingly, upon ns-excitation of rotaxane **1** a long-lived ZnP˙^+^/C_60_˙^–^ charge separated state is formed in addition to the ZnP triplet excited state. Evidence for the transient formation of one electron oxidized ferrocene and/or [Cu(phen)_2_]^+^ is hampered by their very low extinction coefficients.^38–43^ Interactions by energy transfer are unlikely to happen for energetic reasons but cannot be ruled out with certainty.

To dissect the different contributions to the transient absorption spectra on rotaxane **1**, that is, the charge separated states, the ZnP triplet excited state, and the C_60_ triplet excited state, the decay kinetics of the one electron oxidized form of ZnP at 680 nm and the one electron reduced form of C_60_ at 1010 nm were examined in the presence or absence of oxygen. It is well known that oxygen impacts the triplet excited state lifetime of ZnP (Table S3†).^44,45^ We find that the transient at 680 nm decays biexponentially (Fig. S10,† left) with one lifetime that depends strongly on the O_2_ concentration and another one that is nearly constant; 112 μs is the lifetime in the absence of O_2_ and 110 ns under O_2_ saturated conditions (Table S3†), from which a bimolecular quenching rate constant of 1.0 × 10^9^ M^–1^ s^–1^ is derived. The other component has a nearly constant lifetime of 2.3 ± 0.3 μs. The O_2_-dependent lifetime must relate to the ZnP triplet excited state, whereas the component that shows only a weak O_2_ dependence is assigned to the charge separated state involving ZnP˙^+^. Note that the extinction coefficient of the one electron oxidized form of Fc is lower than that of ZnP with a value of 500 M^–1^ cm^–1^.^15^ In light of the aforementioned, we analyzed the decay of C_60_˙^–^ – Fig. S10† – at 1010 nm. From tri-exponential fittings a short lifetime of 55 ± 8 ns as well as an intermediate and a long lifetime of 2.3 ± 0.4 and 61 ± 16 μs were derived. Only the intermediate and long lifetimes exhibit dependence on the concentration of O_2_, yielding bimolecular rate constants of 1.0 × 10^7^ and 1.7 × 10^5^ M^–1^ s^–1^, respectively. Considering that the short lifetime is comparable to that found for **8** ^1,12,18,33^ it is, in turn, assigned to Fc–ZnP–[Cu(phen)_2_]^2+^–C_60_˙^–^. The intermediate lifetime of the 1010 nm decay matches the 680 nm decay, which suggests that it is the Fc–ZnP˙^+^–[Cu(phen)_2_]^+^–C_60_˙^–^ charge separated state. Finally, the long lifetime is assigned to the Fc˙^+^–ZnP–[Cu(phen)_2_]^+^–C_60_˙^–^ long distance charge separated state, since it possesses the lowest energy (1.13 eV).

**Fig. 8:**
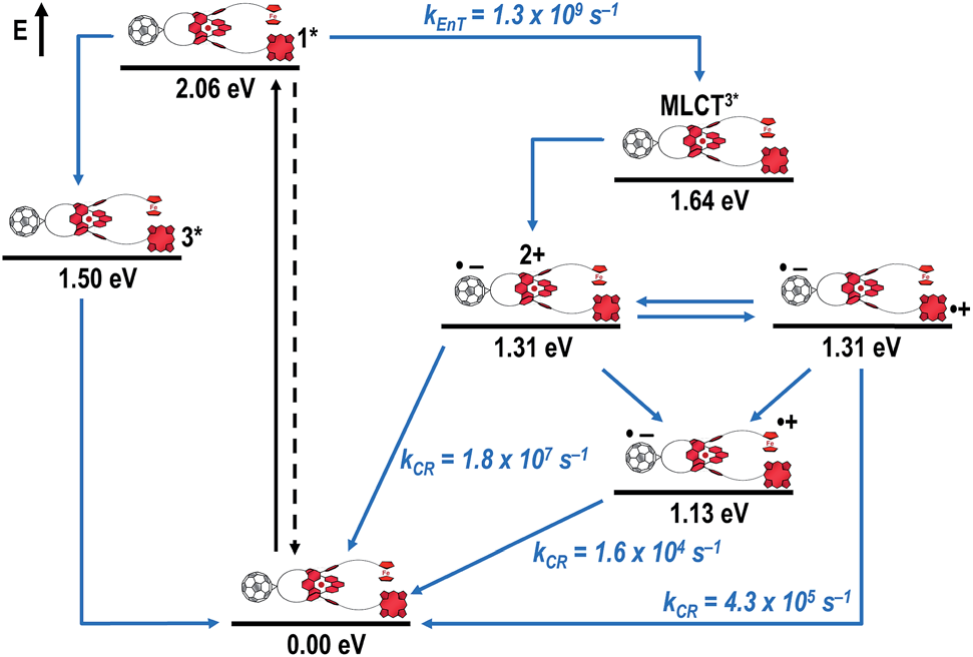
Schematic energy level diagrams, proposed decay pathways, and rate constants for Fc–ZnP–[Cu(phen)_2_]^+^–C_60_ rotaxane **1** upon excitation at 425 nm. *k*_EnT_ = energy transfer rate; *k*_CR_ = charge recombination rate contant.

Fig. 8 schematically summarizes the processes which take place upon 425 nm excitation of rotaxane **1** with the corresponding energy levels calculated from spectroscopic and electrochemical data. Excitation of rotaxane **1** into the ZnP Soret band at 425 nm generates the ZnP singlet excited state with an energy level of 2.06 eV relative to the ground state. From these states, different deactivation pathways emerge. Firstly, a deactivation to the ground state *via* fluorescence occurs with a quantum yield of 2%. Secondly, intersystem crossing (ISC) yields the energetically lower-lying ZnP centered triplet excited state (1.50 eV), which subsequently decays to the ground state. In competition, energy transfer from the ZnP singlet excited state to [Cu(phen)_2_]^+^ takes place. The energy transduction to [Cu(phen)_2_]^+^ is followed by a rapid and unresolvable ISC to ^3^MLCT*. From the latter, electron transfer generates C_60_˙^–^ and [Cu(phen)_2_]^2+^ with an energy level at 1.31 eV above the ground state. Experimental confirmation for this electron transfer hypothesis comes from comparison between the ns transient absorption data for **1** with those obtained for catenane **8**. The lifetime of C_60_˙^–^ of 55 ns (*k*_CR_ = 1.8 × 10^7^ s^–1^) in **1** resembles that found for catenane **8** (100 ns).^11,12,16,33^ Despite the fact that [Cu(phen)_2_]^2+^ cannot be identified in the differential absorption spectra, due to its low extinction coefficient, we are confident about the formation and existence of the Fc–ZnP–[Cu(phen)_2_]^2+^–C_60_˙^–^ charge separated state. From this intermediate state, a charge shift process occurs from [Cu(phen)_2_]^2+^ to ferrocene and/or to ZnP. On longer time scales, two additional lifetimes were derived for the one electron reduced C_60_, one of which matches the lifetime of 2.3 μs (*k*_CR_ = 4.3 × 10^5^ s^–1^) found for the one electron oxidized ZnP at 680 nm. Accordingly, this lifetime is assigned to the Fc–ZnP˙^+^–[Cu(phen)_2_]^+^–C_60_˙^–^ charge separated state, which is apparently isoenergetic with the Fc–ZnP–[Cu(phen)_2_]^2+^–C_60_˙^–^ one, which favors interaction between these two states. The lifetime of 61 μs (*k*_CR_ = 1.6 × 10^4^ s^–1^) is attributed to the Fc˙^+^–ZnP–[Cu(phen)_2_]^+^–C_60_˙^–^ long distance charge separated state. This state is expected to be the thermodynamically most stable state at 1.13 eV relative to the ground state (Fig. 8). This stable state can be generated by two thermodynamically possible charge shift scenarios, that is, one evolving from Fc–ZnP–[Cu(phen)_2_]^2+^–C_60_˙^–^ and the other from Fc–ZnP˙^+^–[Cu(phen)_2_]^+^–C_60_˙^–^. Considering the slow charge recombination in Fc˙^+^–ZnP–[Cu(phen)_2_]^+^–C_60_˙^–^, with a rate constant of *k*_CR_ = 1.6 × 10^4^ s^–1^, it is clear that charge recombination is located in the inverted region of the Marcus parabola.^46,47^

#### 4.2 Rotaxane **2**

Excitation of rotaxane **2** at 660 nm yields the ZnPc singlet excited state identified by the 825 nm transient absorption.^31,43^ The latter deactivates *via* ISC. The ZnPc triplet excited state with its 490 nm marker is stable on the time scale of the experiment. Ground state bleaching is observed at 610 and 680 nm. Additionally, a new peak in the near infrared region at 1010 nm emerges, which is the fingerprint of C_60_˙^–^.^11,12,44^ From this, we conclude that electron transfer takes place in rotaxane **2** in competition with ISC. Excitation into the ZnP Soret band of **2** at 420 nm results in population of the ZnP singlet excited state which decays within 40 ps to give the ZnP triplet excited state as well as the energetically lower lying ZnPc singlet excited state, as found for **6** and **7** (ESI†). In this case, no clear signs for any transduction of excited state energy were found.

**Fig. 9:**
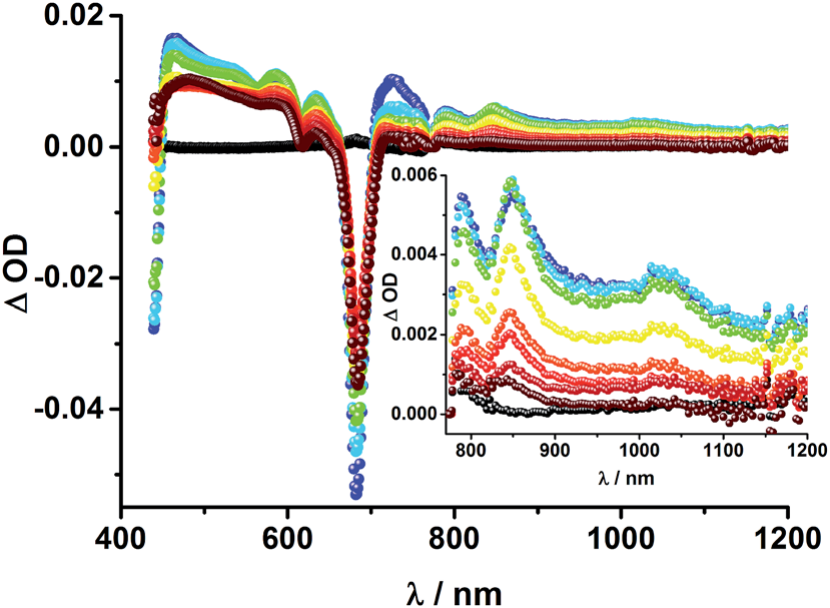
Transient absorption spectrum (visible and near-infrared) registered upon femtosecond flash photolysis (387 nm, 200 nJ) of ZnP–ZnPc–Cu(phen)_2_–C_60_ rotaxane **2** in PhCN with time delays between 0 (black) and 7.5 ns (wine) at room temperature. Inset: enhanced spectra in near infrared region.

Upon excitation of rotaxane **2** at 387 nm, not only the ZnP and ZnPc transient features are observed in the differential absorption spectra, as seen upon 660 and 420 nm excitation, but also those of C_60_ and [Cu(phen)_2_]^+^ (Fig. 9). In the visible region of the spectrum, the C_60_ singlet excited state marker at 510 nm and ^3^MLCT* marker at 600 nm are masked by the more intense ZnP and ZnPc transient absorptions. In the near-infrared region, a rather broad transient absorption is observed that corresponds to the triplet MLCT state. Additionally, two maxima are identified at 850 and 1020 nm in PhCN or 840 and 1020 nm in THF. The 1020 nm transient, the well-known fingerprint of C_60_˙^–^, is stable over the 7.5 ns time scale of our experiment.^48,49^ The 840–850 nm transient corresponds to the ZnPc singlet excited state with a lifetime of 2.2 ns in PhCN and 1.6 ns in THF. Notably, mono-exponential fitting is not sufficient to describe the underlying transient decay. The 850 nm transient, which is stable over the 7.5 ns time scale, can be assigned to the one electron oxidized ZnPc.^50^ Thus, we conclude that electron transfer from ZnPc to C_60_ takes place. The possibility that ZnP is also involved in an electron transfer process cannot be ruled out, since its transient features coincide with the ZnPc ground state bleaching. However, based on thermodynamics we conclude this pathway is very unlikely.

**Fig. 10:**
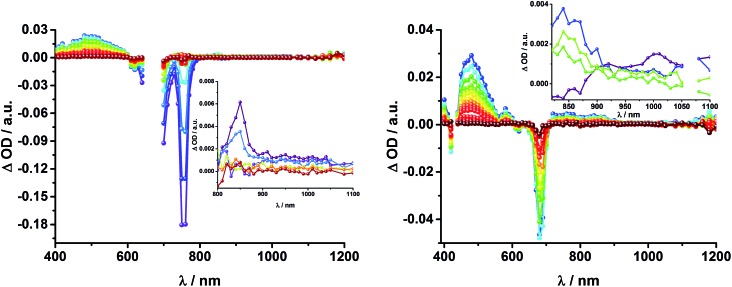
Left: Differential absorption spectra (visible and near infrared) registered upon nanosecond flash photolysis (670 nm, 5 mJ) of ZnP–ZnPc–[Cu(phen)_2_]^+^–C_60_ rotaxane **2** under aerobic conditions in THF with time delays between 90 ns (blue) and 1.0 μs (wine) at room temperature. Inset: zoom into the near infrared region with time delays between 65 ns (purple) and 1.0 μs (wine). Right: Differential absorption spectra (visible and near infrared) registered upon nanosecond flash photolysis (425 nm, 5 mJ) of ZnP–ZnPc–[Cu(phen)_2_]^+^–C_60_ rotaxane **2** under aerobic conditions in THF with time delays between 120 ns (blue) and 4.0 μs (wine) at room temperature. Inset: expanded spectra in the near infrared region with time delays between 23 ns (purple) and 400 ns (green).

In complementary ns-transient absorption experiments, rotaxane **2** was excited into the ZnP Soret band at 425 nm and into the ZnPc Q-band at 670 nm. Upon ZnPc excitation, the visible region of the differential absorption spectra is dominated by the broad ZnPc triplet excited state signature at 480 nm (Fig. 10, left). The latter is oxygen sensitive, with lifetimes of ∼300 ns in the presence of oxygen and ∼14 μs in the absence of oxygen. Absorption signatures corresponding to C_60_ triplet excited state and/or ^3^MLCT* were not observed due to the intense ZnPc ground state bleaching between 650 and 750 nm. In the near-infrared region, two maxima are discernible at 850 and 1010 nm. The former is assigned to the one-electron oxidized ZnPc, which decays with a lifetime of ∼520 ± 100 ns, while the latter is the one-electron reduced C_60_ and exhibits two decay lifetimes, 64 ± 26 ns and 570 ± 110 ns. The shorter lifetime matches those found for reference compound **8** ^11,12,16,33^ and the close charge separated states in rotaxane **1**. Thus, it is assigned to the [Cu(phen)_2_]^+^ centered charge separated state ZnP–ZnPc–[Cu(phen)_2_]^2+^–C_60_˙^–^. The longer lifetime is in agreement with the ZnP–ZnPc˙^+^–[Cu(phen)_2_]^+^–C_60_˙^–^ long distance charge separated state. No indication of a charge shift to form ZnP˙^+^–ZnPc–[Cu(phen)_2_]^+^–C_60_˙^–^ was seen upon 670 nm excitation.

**Fig. 11:**
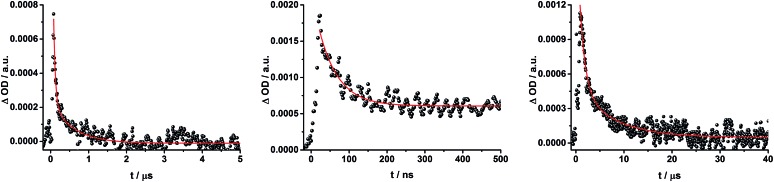
Time absorption profiles at 1010 nm of ZnP–ZnPc–[Cu(phen)_2_]^+^–C_60_ rotaxane **2** in THF at room temperature under aerobic conditions upon excitation at 670 nm (left) and 425 nm (center and right), monitoring the charge recombination.

**Fig. 12:**
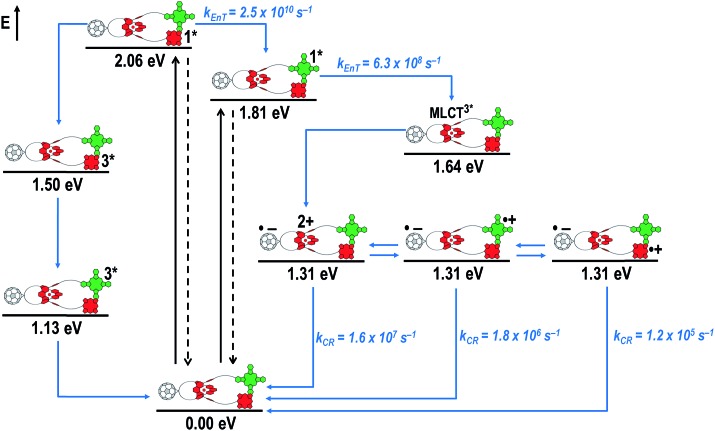
Schematic energy level diagrams, proposed decay pathways, and rate constants for ZnP–ZnPc–[Cu(phen)_2_]^+^–C_60_ rotaxane **2** upon excitation at 425 nm and 670 nm. *k*_EnT_ = energy transfer rate *k*_CR_ = charge recombination rate constant.

When exciting into the ZnP Soret band at 425 nm, the transient absorption spectra are slightly different (Fig. 10, right). Here, the dominant features belong to those of ZnP with maxima at 480 and 840 nm. The biexponential decay of the 480 nm transients, gives lifetimes corresponding to the triplet excited state of ZnP (140 μs under Ar) as well as that of ZnPc (14 μs under Ar). Thus, it is reasonable to assume that upon excitation of ZnP two deactivation pathways occur; ISC to the triplet manifold and energy transfer to ZnPc followed by ISC. This observation is also in agreement with the fs transient absorption data. In addition to the strongly oxygen dependent ZnPc triplet excited state lifetime, the decay at 840 nm yields a second component with a lifetime of 560 ± 100 ns. This lifetime correlates with that seen for ZnPc˙^+^, as seen upon 670 nm excitation. Furthermore, the decay of C_60_˙^–^ at ∼1010 nm can be fit with three lifetimes, 63 ± 16 ns, 590 ± 150 ns, and 8.4 ± 1.0 μs (Fig. 11). Again, the shortest lifetime is assigned to ZnP–ZnPc–[Cu(phen)_2_]^2+^–C_60_˙^–^. In contrast to the 670 nm excitation, in this case two different charge shifts seem to occur. Firstly, the ZnPc centered charge separated state ZnP–ZnPc˙^+^–[Cu(phen)_2_]^+^–C_60_˙^–^ with a lifetime of 560 ± 120 ns (from 840 and 1010 nm decays) is formed. Secondly, the longer lived ZnP centered charge separated state ZnP˙^+^–ZnPc–[Cu(phen)_2_]^+^–C_60_˙^–^ with a lifetime of 8.4 μs is generated. The corresponding cation again cannot be identified, since it coincides with the ZnPc ground state bleaching in the 680 nm region. Overall, these results are in agreement with the transient lifetimes found for rotaxane **1**. A schematic energy level diagram of the photoinduced processes in rotaxane **2** is shown in Fig. 12, including energy levels calculated from electrochemical and spectroscopic data. Two different excitation routes are feasible, which result in slightly different deactivation processes. When exciting ZnPc at 670 nm, its singlet excited state is immediately formed, with an energy level of 1.81 eV relative to the ground state. Deactivation *via* fluorescence (12%) as well as *via* ISC takes place. In parallel, energy transfer from the ZnPc singlet excited state to [Cu(phen)_2_]^+^ occurs, which is verified by ∼80% quenching of the ZnPc fluorescence (Table S2†). Next, the ^1^*MLCT undergoes rapid ISC to give ^3^MLCT* (1.64 eV). By analogy to **1**, the ^3^MLCT* decays through electron transfer to yield the charge separated state ZnP–ZnPc–[Cu(phen)_2_]^2+^–C_60_˙^–^. The rate constant for the charge recombination of ZnP–ZnPc–[Cu(phen)_2_]^2+^–C_60_˙^–^ is 1.6 × 10^7^ s^–1^ (63 ns), in the same range as that observed for **8** ^11,12,18,33^ and **1**. A charge shift from the oxidized [Cu(phen)_2_]^2+^ to ZnPc yields the charge separated state ZnP–ZnPc˙^+^–[Cu(phen)_2_]^2+^–C_60_˙^–^, whose formation is corroborated by the observation of the same lifetimes (560 ns, *k*_CR_ = 1.8 × 10^6^ s^–1^) for the one electron oxidized ZnPc and the one electron reduced C_60_. No proof for the formation of the ZnP centered charge separated state was found upon 670 nm excitation. However, when exciting rotaxane **2** at 425 nm, the ZnP singlet excited state (2.06 eV) is formed and additional decay pathways emerge. On one hand, the ZnP triplet excited state (∼1.5 eV) is generated *via* ISC, which decays back to the ground state. On the other hand, energy transfer to the energetically lower lying ^1^*ZnPc (1.81 eV) takes place, which has been observed by fluorescence as well as excitation spectra (Fig. 4 and Table S2†). From this point on, the same processes occurring upon excitation at 670 nm take place. However, when taking a closer look at C_60_˙^–^ decay at 1010 nm, a third much longer lifetime of 8.4 μs (*k*_CR_ = 1.2 × 10^5^ s^–1^) was identified. Consequently, we conclude that upon excitation of rotaxane **2** at 425 nm, a second charge shift from ZnP–ZnPc˙^+^–[Cu(phen)_2_]^2+^–C_60_˙^–^ takes place to form the ZnP centered long distance charge separated state ZnP˙^+^–ZnPc–[Cu(phen)_2_]^2+^–C_60_˙^–^. Considering the energy of the three different charge separated states relative to the ground state, it must be noted that they exhibit approximately the same energy level (1.31 eV), since the oxidation potentials of [Cu(phen)_2_]^+^, ZnP, and ZnPc do not differ appreciably.

#### 4.3 Rotaxane **3**

**Fig. 13:**
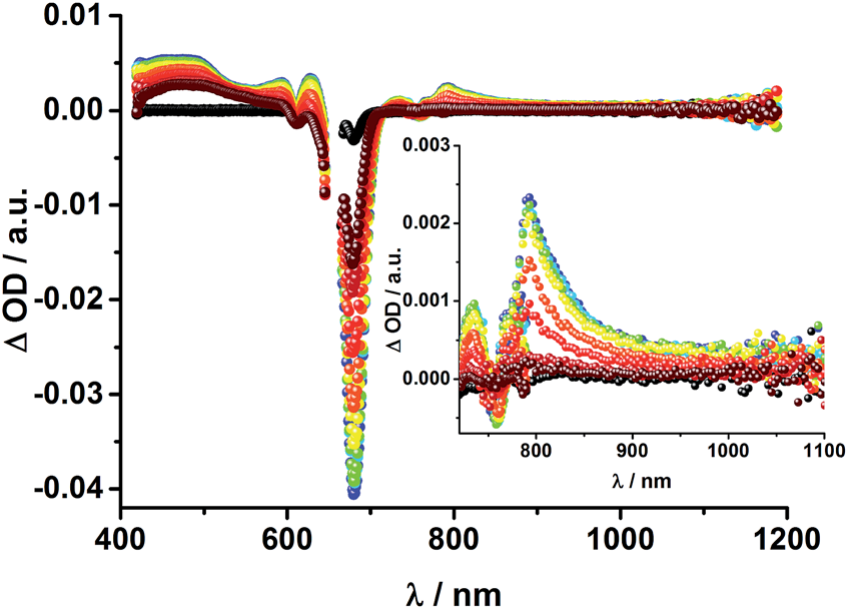
Transient absorption spectrum (visible and near-infrared) registered upon femtosecond flash photolysis (660 nm, 150 nJ) of Fc–ZnPc–[Cu(phen)_2_]^+^–C_60_ rotaxane **3** in THF with time delays between 0 (black) and 5.8 ns (wine) at room temperature. Inset: zoom into the near infrared region.

Finally, rotaxane **3** was probed with our fs transient absorption setup. The 660 nm fs-excitation exclusively excites ZnPc. Immediately after the laser pulse the ZnPc singlet excited state arises with a maximum at 790 nm and a lifetime of 1.9 ns in THF (800 nm, 1.9 ns in PhCN) (Fig. 13). The singlet excited state decays *via* intersystem crossing (ISC) to the energetically lower lying triplet excited state (∼480 nm), which is stable over the time scale of our experimental setup (7.5 ns). Additionally, ground state bleaching leads to minima at 610 and 680 nm in THF and 615 and 690 nm in PhCN. No clear assignment of transients corresponding to C_60_, [Cu(phen)_2_]^+^, or any charge separated state could be made in the fs transient absorption experiments, due to their low extinction coefficients and the relatively low energy of the laser excitation (150 nJ). Weak, but broad transient absorptions in the near infrared region (Fig. 13, inset) allows us to conclude that energy transfer occurs to yield the ^3^MLCT* state.

**Fig. 14:**
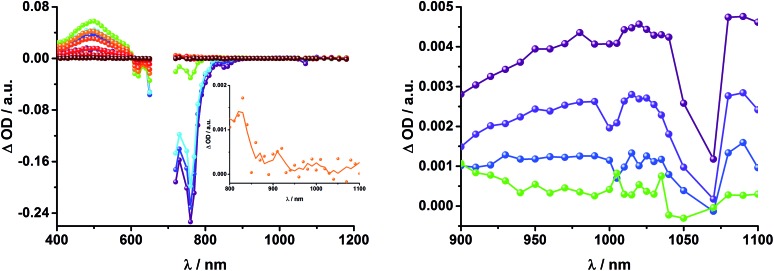
Left: Differential absorption spectra (visible and near infrared) registered upon nanosecond flash photolysis (670 nm, 5 mJ) of Fc–ZnPc–[Cu(phen)_2_]^+^–C_60_ rotaxane **3** under aerobic conditions in THF with time delays between 9 ns (purple) and 2.0 μs (wine) at room temperature. Inset: zoom into the near infrared region after 200 ns. Right: Differential absorption spectra (near infrared) registered upon nanosecond flash photolysis (670 nm, 5 mJ) of Fc–ZnPc–[Cu(phen)_2_]^+^–C_60_ rotaxane **3** under aerobic conditions in THF with time delays between 9 (purple) and 50 ns (green) at room temperature.

**Fig. 15:**
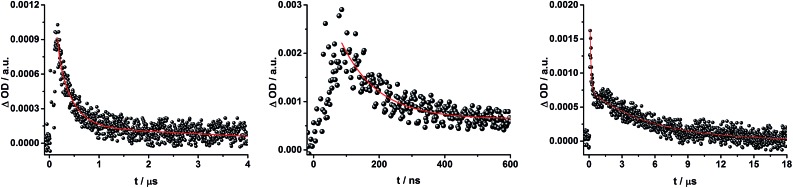
Time absorption profiles of Fc–ZnPc–[Cu(phen)_2_]^+^–C_60_ rotaxane **3** upon 670 nm excitation in THF at room temperature under aerobic conditions at 830 nm (left) and 1010 nm (center and right), monitoring the charge recombination.

To gain further insight into the charge transfer dynamics, we turned to longer time scales. Upon ns-excitation (note that in the ns experiment the laser energy is significantly higher than in the fs experiments) of the ZnPc Q-band at 670 nm, the visible region of the differential absorption spectra is dominated by the triplet excited state signature of ZnPc with its maximum at 500 nm, accompanied by ground state bleaching at 610 and 690 nm (Fig. 14, left).^51–57^ In the near-infrared region (Fig. 14), the broad ^3^MLCT* absorption (∼900–1000 nm) is observed. Additionally, two peaks are discernible at 830 and 1020 nm. The former is assigned to ZnPc˙^+^, which decays mono-exponentially with a lifetime of 380 ± 60 ns (Fig. 15, left). The peak at 1020 nm corresponds to the one-electron reduced C_60_ and it is best fit with three exponentials, as seen earlier for rotaxanes **1** and **2** (Fig. 15, center and right). The shortest lifetime of 88 ± 9 ns resembles that found in catenane **8** ^11,12,18,33^ and is ascribed to the [Cu(phen)_2_]^+^ centered charge separated state Fc–ZnPc–[Cu(phen)_2_]^2+^–C_60_˙^–^. The intermediate lifetime of 380 ± 90 ns matches the lifetime of the one electron oxidized ZnPc and consequently correlates with the ZnPc-centered charge separated state Fc–ZnPc˙^+^–[Cu(phen)_2_]^+^–C_60_˙^–^. Finally, the longest lifetime associated with C_60_˙^–^ of 4.9 ± 0.7 μs is assigned to the thermodynamically most stable long distance charge separated state, namely Fc˙^+^–ZnPc–[Cu(phen)_2_]^+^–C_60_˙^–^. This is consistent with the results obtained for rotaxane **1**.

**Fig. 16:**
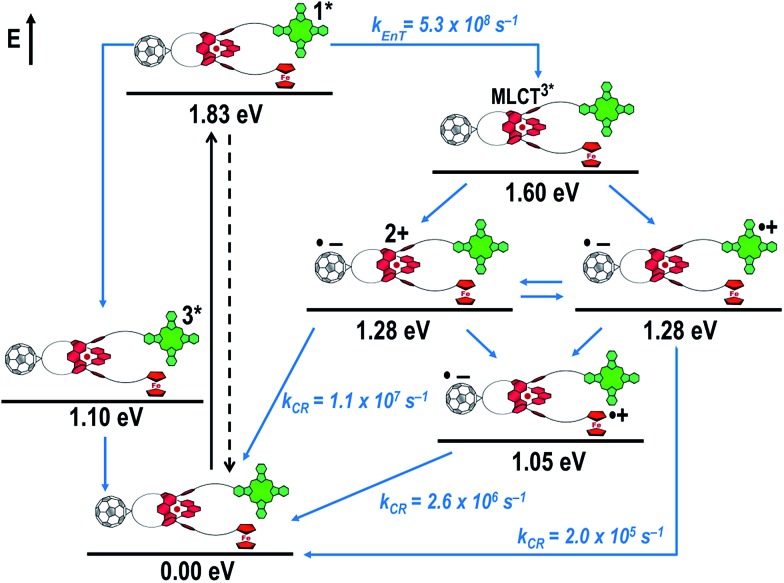
Schematic energy level diagrams, proposed decay pathways, and rate constants for Fc–ZnPc–[Cu(phen)_2_]^+^–C_60_ rotaxane **3** upon excitation at 670 nm. *k*_EnT_ = energy transfer rate *k*_CR_ = charge recombination rate constant. Energy levels not to scale.

Fig. 16 schematically outlines the excitation and deactivation pathways in rotaxane **3**. When exciting directly into the ZnPc Q-bands at 670 nm or into the ZnPc Soret band at 387 nm, the ZnPc singlet excited state is generated, with an energy of 1.83 eV relative to the ground state. Deactivation processes include fluorescence with a quantum yield of 15% and ISC to give the triplet excited state of ZnPc (∼1.1 eV) followed by the triplet signature in the ns-transient absorption near 500 nm. Deactivation occurs *via* energy transfer to [Cu(phen)_2_]^+^, which is confirmed by 50% quenching of the ZnPc emission (Table S2†). Spectroscopically only the ^3^MLCT* (∼1.6 eV) state can be resolved, since the ^1^MLCT* is only stable for several hundreds of femtoseconds.^58,59^ Electron transfer takes place from the ^3^MLCT* state to generate the charge separated state Fc–ZnPc–[Cu(phen)_2_]^2+^–C_60_˙^–^ with a lifetime of 88 ns (*k*_CR_ = 1.1 10^7^ s^–1^). This state has been identified from the signature absorption of C_60_˙^–^ at 1010 nm (Fig. 14 and 15). Furthermore, two additional lifetimes were found for the C_60_˙^–^. Thus, we conclude that from the state Fc–ZnPc–[Cu(phen)_2_]^2+^–C_60_˙^–^ a charge shift occurs to form the ZnPc- and Fc-centered charge separated states, which are stable for 380 ns (*k*_CR_ = 2.6 × 10^6^ s^–1^) and 4.9 μs (*k*_CR_ = 2.0 10^5^ s^–1^), respectively. From the present data, it has been impossible to verify with certainty whether the two charge shift processes take place simultaneously or successively. The assignment of the lifetimes from the 1010 nm decay has been made by comparison with ZnPc˙^+^ decay at ∼830 nm, and by comparison with the charge separated state lifetimes found for rotaxanes **1** and **2**. Considering the energy levels of the three different charge separated states, Fc–ZnPc–[Cu(phen)_2_]^2+^–C_60_˙^–^ and Fc–ZnPc˙^+^–[Cu(phen)_2_]^+^–C_60_˙^–^ seem to exhibit the same energy level, namely 1.28 eV, while the Fc˙^+^–ZnPc–[Cu(phen)_2_]^+^–C_60_˙^–^ long distance charge separated state at 1.05 eV is thermodynamically most stable.

## Conclusion

It is evident that all three of the newly investigated donor–acceptor rotaxanes undergo similar deactivation pathways upon photoexcitation. The differences become obvious when looking at the charge separated state lifetimes. The [Cu(phen)_2_]^+^ centered charge separated states feature lifetimes shorter than 100 ns, while the corresponding ZnPc-centered charge separated state in rotaxanes **2** and **3** exhibit lifetimes in the range of several hundreds of nanoseconds. Although the ZnP centered CS state is energetically at a similar level as those centered at the [Cu(phen)_2_]^+^ complex and ZnPc (∼1.3 eV), considerably longer lifetimes (in the microsecond time regime) are observed for the former. In case of the [Cu(phen)_2_]^+^-centered charge separated sate, the shorter lifetimes arise from shorter distances between the radical ions, while ZnPc is generally known to form shorter lived radical ion pairs with C_60_ than ZnP, since smaller energy gaps for the back electron transfer step pulls these processes closer to the top of the Marcus parabola.^1,60^ The longest lifetimes determined are for the energetically favored Fc-centered charge separated state (∼1.1 eV). However, in rotaxane **1**, the lifetime (61 μs) is considerably longer than for rotaxane **3** (4.9 μs). Thus, it is concluded that the Fc˙^+^–C_60_˙^–^ charge separated state is better stabilized by ZnP (rotaxane **1**) than by ZnPc (rotaxane **3**).

In comparison to previously studied rotaxanes incorporating either ZnPc,^61^ Fc,^16^ or ZnP,^14,15,18^ significantly longer charge separated state lifetimes have been achieved in the new rotaxanes **1**, **2**, and **3**. For example, in ferrocene stoppered [Cu(phen)_2_]^+^–C_60_ rotaxanes only the (Fc)_2_–[Cu(phen)_2_]^2+^–C_60_˙^–^ charge separated state with a lifetime of 15–16 ns (*k*_CR_ = 6.3 to 6.7 × 10^7^ s^–1^) was observed, without any appreciable evidence for a subsequent charge shift to the ferrocene units.^16^ However, when replacing one of the Fc stoppers by ZnP (rotaxane **1**) or ZnPc (rotaxane **3**), a charge shift from ZnP and ZnPc to the ferrocene takes place to yield the thermodynamically most stable Fc-centered charge separated states, with lifetimes in the microsecond regime. In our previously reported C_60_-stoppered porphyrino-rotaxanes, a long lived ZnP˙^+^–[Cu(phen)_2_]^+^–(C_60_)_2_˙^–^ of 32 μs lifetime (*k*_CR_ = 3.1 × 10^4^ s^–1^) were detected.^14^ However, when combining the virtues of ZnP and Fc as electron donors, as in the present work, charge separated states with almost twice that lifetime are achievable. Therefore, the combination in a single [Cu(phen)_2_]^+^-based rotaxane system of the outstanding electrochemical and spectroscopic properties of ZnP with the low oxidation potential of ferrocene and the extraordinary electron accepting properties and low reorganization energy of C_60_ yields very attractive photoactive materials, which are capable of absorbing light over a very wide range of the visible spectrum and undergoing a cascade of energy and electron transfer processes that ultimately produce charge separated states with lifetimes in the microsecond time domain, as high as 61 μs.
