# Supplementary material for: Multistep energy and electron transfer processes in novel rotaxane donor–acceptor hybrids generating microsecond-lived charge separated states
Source: Chem Sci. 2015 Oct 2;6(12):7293–304. doi: 10.1039/c5sc02895g (PMC5512142; doi:10.1039/c5sc02895g)
Supplement: Supplementary file 1 [file SC-006-C5SC02895G-s001.pdf]

Electronic Supplementary Material (ESI) for Chemical Science.
This journal is © The Royal Society of Chemistry 2015

Electronic Supporting Information

Multistep Energy and Electron Transfer Processes in Novel Rotaxane Donor–Acceptor Hybrids Generating Microsecond Lived Charge Separated States

Sabrina V. Kirner,[a] Christian Henkel[a], Dirk M. Guldi,*[a] Jackson D. Megiatto, Jr.[b†] and David I. Schuster*[b]

[a] Department of Chemistry and Pharmacy and Interdisciplinary Center for Molecular Materials, Friedrich-Alexander-Universität Erlangen-Nürnberg, Egerlandstrasse 3, 91058 Erlangen, Germany

[b] Department of Chemistry, New York University, New York, *NY 10003, USA.*

[†] Present Address: Institute of Chemistry, University of Campinas, PO Box 6154, Campinas, SP, 13084-861, Brazil.

1. Supplementary Figures 2
2. Structural Characterization 3
2.1. MALDI-TOF Mass Spectrometry 3
3. Ground State Interactions 5
3.1. Electrochemistry 5
3.2 UV-vis Absorption 6
4. Excited State Interactions 8
4.1. Emission Spectroscopy 8
4.2. Transient Absorption 12
4.2.1. References excited at 387 nm (fs-laser) and 355 nm (ns-laser) 13
4.2.2. References excited at 420 nm (fs-laser) 13
4.2.3. References excited at 387 / 420 nm (fs-laser) and 425 nm (ns-laser) 14
4.2.4. References excited at 387 / 420 / 660 nm (fs-laser) and 425 / 670 nm (ns-laser) 15
5. Experimental Section 18
5.1. General Information and Materials 18
5.2. Synthesis 19
5.3. Electrochemical and Photophysical Studies 22
6. Literature 23

1. Supplementary Figures

Scheme S1 Convergent synthetic strategy adopted to prepare target rotaxanes **1**, **2**, and **3**.

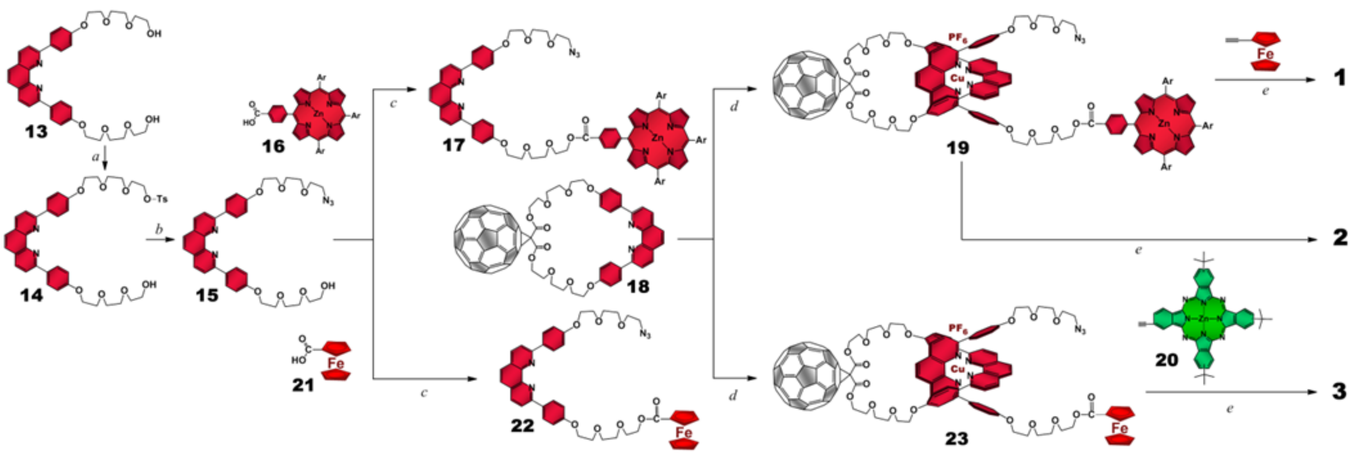

(a) tosyl chloride, dichloromethane, 0°C for 4 h then rt for 12 h, 45% yield; *(b)* sodium azide, dimethylformamide, 80°C, 12 h, 90% yield; *(c)* 1-ethyl-3-(3-dimethylaminopropyl)carbodiimide (EDC), dimethylaminopyridine (DMAP), dichloromethane, rt, 12 h, 85% yield; *(d)* $Cu(CH_3CN)_4][PF_6]$, dichloromethane/acetonitrile (7:3, v/v), rt, 12 h, quantitative (by TLC); *(e)* copper iodide, sodium ascorbate, sulfonated bathophenathroline, 1,8-Diazabicycloundec-7-ene (DBU), dichloromethane/water/ethanol (1:1:1, v/v), 70°C, 12 h. Ar = 3,5-di-*tert*-butylphenyl.

2. Structural Characterization

2.1. MALDI-TOF Mass Spectrometry

MALDI-TOF analysis in positive mode for all rotaxanes revealed the expected ion mass peak corresponding to the molecular mass of the rotaxanes lacking the PF_6^- counter-ion as well as the classical fragmentation pattern observed for $[Cu(phen)_2]^+$-based interlocked systems upon ionization.[1-4] For example, a MALDI-TOF spectrum of rotaxane **2** (Figure S1) clearly reveals the ion mass peak at m/z 3939.9 (rotaxane **2** - PF_6) corresponding to the proposed rotaxane structure. The latter comes along with two extra ion mass peaks both with high intensity. These peaks correspond to a fragment lacking the fullerene (m/z 3219.4), suggesting rupture of the cyclopropane moiety that links the carbon cage to the phen-macrocycle, and to the thread component coordinated to a Cu ion (m/z 2522.8), indicating further rupture and loss of the phen-macrocycle.

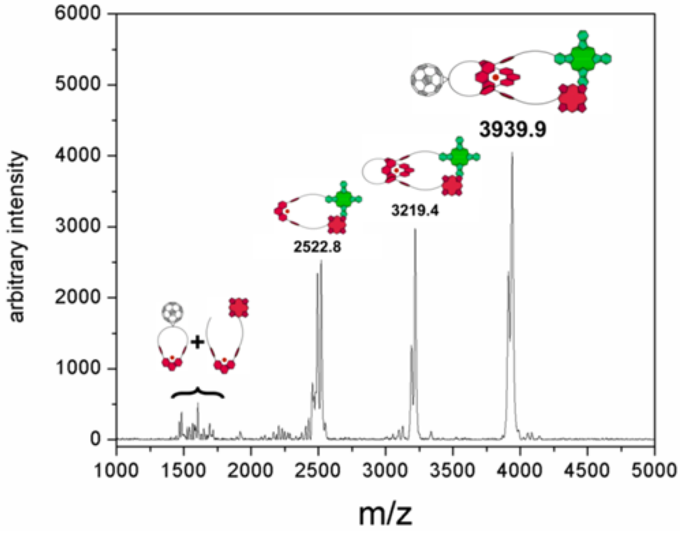

Fig. S1: MALDI-TOF mass spectrum of rotaxane **2**. Positive mode, α-cyano-4-hydroxycinnamic acid (CCA) used as matrix.

It is interesting to note that the ion mass peak at m/z 3219.4 appears as doublets, while the peak at m/z 2522.8 appears as a triplet. The doublets have a difference of m/z 28, while the triplet signal has a difference of m/z 28 and 62. We propose that the extra peaks that appear at m/z 28 units lower in the MALDI spectrum correspond to species that had lost a single N_2 molecule due to fragmentation of the triazole ring upon ionization.[5, 6] The species detected at m/z 62 units lower corresponds to the protonated thread fragment lacking the Cu ion. The minor peaks in the MALDI spectrum likely correspond to unusual fragments of the rotaxane structure since increasing the laser power during the MALDI experiments increases the intensity of those minor peaks while

significantly decreasing the peak corresponding to rotaxane **2**. Similar MALDI spectra were obtained for rotaxanes **1** and **3**.

2.2. ^{1}H Nuclear Magnetic Resonance – NMR

^{1}H NMR investigation revealed that rotaxanes **1**, **2**, and **3** are very flexible structures existing in several interconverting conformations with complex dynamics on the NMR time scale. It is safe to assume that they are driven by secondary interactions between the chromophores.[1, 2, 7-13] Variable temperature ^{1}H NMR studies on rotaxane **1** in DMF-d_7 showed increasing broadening of the peaks at lower temperatures with no coalescence even at temperatures as low as 233 K. A somewhat cleaner spectrum was obtained at 100°C, which allowed us to identify key protons of the proposed structure, namely the pyrrolic protons of the porphyrin between 8.92 and 8.86 ppm, the triazole proton at 8.05 ppm, and the ferrocene nuclei[10, 14] at 4.71, 4.33 and 4.01 ppm as well as the protons attached to the phenyl rings linked to positions 2 and 9 of the phen moieties, which appears in the usual upfield region at 7.42, 7.22, 6.18, 6.09 ppm, confirming that the two phen groups are entwined around the Cu(I) template ion.[15] For rotaxanes **2** and **3**, which bear phthalocyanines, aggregation is evident in the ^{1}H NMR spectra even at high temperatures. Although some key features of the proposed rotaxane structures can be safely assigned, the signals are unfortunately too broad for a detailed analysis.

3. Ground State Interactions

3.1. Electrochemistry

To probe the redox properties of the electron donor-acceptor rotaxanes (**1**-**3**) as well as the corresponding reference compounds (**4**-**12**) square wave voltammetry and differential pulse voltammetry experiments were carried out in dichloromethane (DCM) in the presence of 0.1 M tetrabutylammonium hexafluorophosphate ($TBAFPF_6$) as supporting electrolyte and ferrocene/ferrocenium as internal reference. Table S1 summarizes the electrochemical data with all redox potentials reported in volts (V) relative to the ferrocene couple (Fc/Fc+).

While the C_{60} reference **10** was inactive under oxidative conditions, one-electron reductions were observed at −1.06 and −1.44 V resembling the trend found for pristine C_{60}.[16-18] Due to the partial loss of π-conjugation, **10** reveals a shift towards more negative reductions when compared to pristine C_{60}. ZnTPP (**11**) exhibits one-electron oxidations at +0.28 and +0.62 V. $Zn^{t}Bu_4Pc$ (**12**) features only one oxidation at +0.22 eV within the electrochemical window. Catenane **9** reveals a single oxidation at +0.16 V, which correlates with the one-electron oxidation of the copper center, namely $[Cu(phen)_2]^{+}/[Cu(phen)_2]^{2+}$.[1, 7] C_{60}-$[Cu(phen)_2]^{+}$ catenane **8** shows two one electron reductions at −1.12 and −1.48 V, corresponding to C_{60} centered processes. In addition, the one-electron oxidation of $[Cu(phen)_2]^{+}$ evolves at +0.16 V. To this end, the presence of C_{60} has no notable impact on the $[Cu(phen)_2]^{+}$ oxidation. Reference thread **5** features two oxidations at +0.26 and +0.62 V, corresponding to the oxidation of ZnP. A third oxidation at -0.01 V assigned to the ferrocene oxidation is shifted by 0.01 V to lower potentials compared to the ferrocene reference. In rotaxane **4**, three oxidations are discernible. The first oxidation at +0.03 V is assigned to a ferrocene centered process. The second oxidation at +0.28 V is twice in intensity when compared to the one at +0.03 V. Thus, we hypothesize that it corresponds to the coalescence of a $[Cu(phen)_2]^{+}$ as well as a ZnP oxidation.[1] Finally, the third oxidation at +0.88 V, which relates to the second oxidation of ZnP, is shifted towards more positive potentials when compared to ZnTPP and reference **11**. It is likely that the presence of $[Cu(phen)_2]^{+}$ impacts the ZnP oxidation. Thread reference **7** is inactive under reductive conditions. Under oxidative conditions, two oxidations are discernible for **7** at +0.21 and +0.75 V. Considering that the former is broader and more intense, we assume that it is a superimposition of the first ZnP oxidation and the first ZnPc oxidation. The second oxidation is assigned to the second oxidation of ZnP. A similar conclusion is derived for reference **6**. Here, the first oxidations of ZnP, ZnPc, and $[Cu(phen)_2]^{+}$ cannot be clearly resolved at about +0.20 V, while the second ZnP oxidation sets in at ~0.7 V. No reduction peaks were observed within the electrochemical window of DCM.

Table S1 Oxidation and reduction potentials. All redox potentials reported in volts (V) are relative to the ferrocene couple (Fc/Fc^+) as internal reference. Electrolyte: 0.1 M $TBAFPF_6$ in dichloromethane (DCM) or *ortho*-dichlorobenzene (*o*-DCB). P = porphyrin and Pc = phthalocyanine.

	**Solvent**	**Oxidation**					**Reduction**	
		$P^{•+}/P^{2+}$	$P/P^{•+}$	$Pc/Pc^{•+}$	Cu^+/Cu^{2+}	Fc/Fc^+	$C_{60}/C_{60}^{•-}$	$C_{60}^{•-}/C_{60}^{2-}$
**1**	DCM	0.88	0.24	–	0.24	0.04	–1.09	–1.45
**2**	DCM	20	0.18	0.18	0.13	–	–1.13	–1.54
**3**	DCM	–	–	0.18	0.18	–0.05	–1.10	–1.55
**4**	DCM	0.88	0.28	–	0.28	0.03	–	–
**5**	DCM	0.61	0.26	–	–	–0.01	–	–
**6**	DCM	0.74	0.20	0.20	0.20	–	–	–
**7**	DCM	0.75	0.21	0.21	–	–	–	–
**8**[1]	*o*-DCB	–	–	–	0.16	–	–1.12	–1.48
**9**[10]	*o*-DCB	–	–	–	0.16	–	–	–
**10**	DCM	–	–	–	–	–	–1.06	–1.44
**11**	*o*-DCB	0.62	0.28	–	–	–	–	–
**12**	DCM	–	–	0.22	–	–	–	–

3.2 UV-vis Absorption

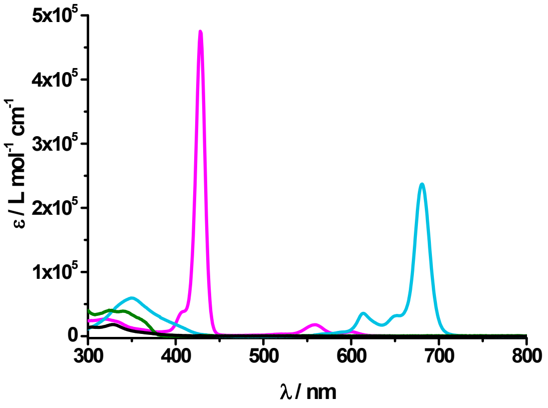

Fig. S2 UV/Vis absorption spectra of reference compounds **9** (olive), C_{60} reference **10** (black), ZnTPP **11** (magenta) and ZnPc **12** (cyan) in PhCN.

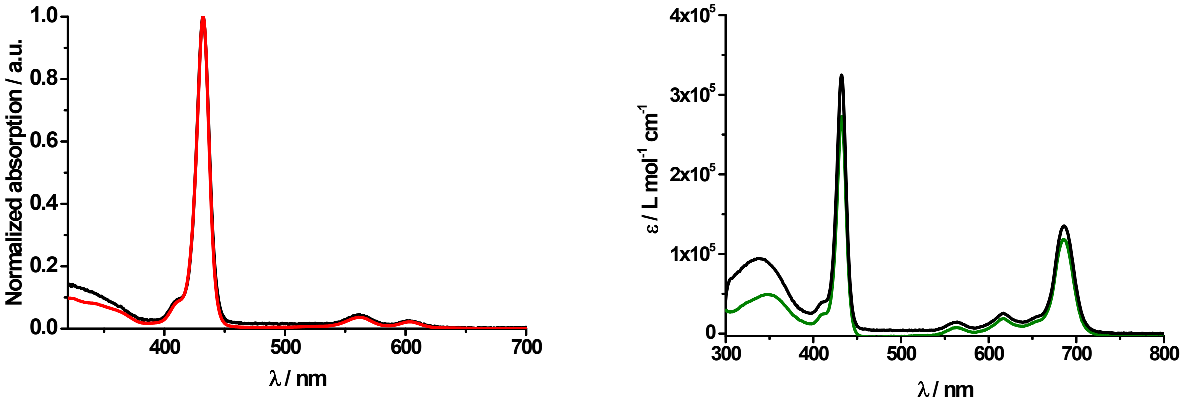

Fig. S3 Left: normalized UV/Vis absorption spectra of reference compounds **4** (black) and **5** (red) in PhCN. Right: UV/Vis absorption spectra of reference compounds **6** (black) and **7** (olive) in PhCN.

4. Excited State Interactions

4.1. Emission Spectroscopy

Table S2 Fluorescence parameters and fluorescence lifetimes in THF at 298 K in air-equilibrated solutions.[a]

Compound	λ_{exc} (nm)	Excited State	λ_{max} (nm)	τ (ns)	Φ_F	E_{00} (eV)
**1**	420	Fc–**ZnP***–$[Cu(phen)_2]^+$–C_{60}	602	0.82	0.02	2.06
	320	Fc–ZnP**–$[Cu(phen)_2]^{+}$*-**C_{60}	755	–	$3.6x10^{-4}$	1.64
**2**	420	**ZnP***-ZnPc–$[Cu(phen)_2]^+$–C_{60}	602	< 0.15	0.006	2.06
	610	ZnP–**ZnPc***–$[Cu(phen)_2]^+$–C_6	686	1.7	0.12	1.81
**3**	610	Fc–**ZnPc***–$[Cu(phen)_2]^+$–C_{60}	679	2.1	0.15	1.83
**4**	420	Fc–**ZnP***–$[Cu(phen)_2]^+$	604	0.81	0.02	2.05
	320	Fc-ZnP**–$[Cu(phen)_2]^{+}$***	756	–	$7.5x10^{-4}$	1.64
**5**	420	Fc-**ZnP***-(phen)	603	1.8	0.04	2.06
**6**	420	**ZnP*-**ZnPc–$[Cu(phen)_2]^+$	603	0.19	0.008	2.06
	610	ZnP-**ZnPc***–$[Cu(phen)_2]^+$	688	1.6	0.07	1.80
**7**	420	**ZnP***-ZnPc–(phen)	602	0.15	0.004	2.06
	610	ZnP-**ZnPc***–(phen)	686	2.3	0.13	1.81
**8**	320	**$[Cu(phen)_2]^{+}$*-**C_{60}	765	–	$2.5x10^{-4}$	1.62
	350	$[Cu(phen)_2]^+$-**C_{60}***	710	–	$2.3x10^{-5}$	1.75
**9**[b]	320	**$[Cu(phen)_2]^{+}$***	759	2.5	$4.8x10^{-3}$	1.63
**10**	350	**$^{1*}C_{60}$-malonate**[d]	715	1.48	$1.0x10^{-3}$	1.73
**11**	420	**ZnTPP***	602	2.0	0.04	2.06
**12**	610	**ZnPc***	679	3.7	0.3	1.83

[a] λ_{max} = emission maxima; τ = fluorescence lifetime; Φ_F = fluorescence quantum yields; E_{00} = Energy of the corresponding excited state relative to the ground state, calculated from the emission maximum (λ_{max}); [b] from reference 3.

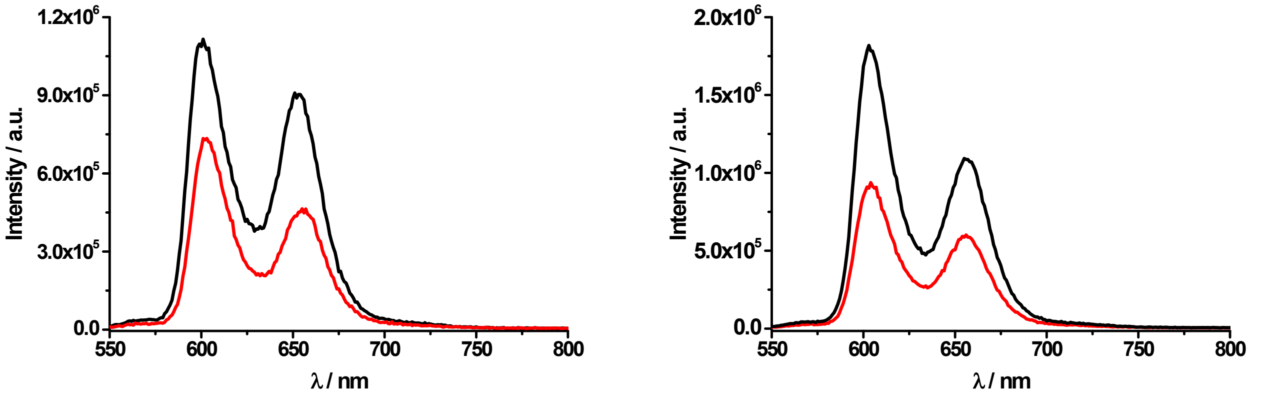

Fig. S4 Left: emission spectra of Fc-ZnP-$[Cu(phen)_2]^+$-C_{60} **1** (red) and ZnTPP **11** (magenta) in THF upon excitation at 420 nm (OD = 0.045). Right: Emission spectra of reference compounds **4** (red) and **5** (black) in THF upon excitation at 420 nm (OD = 0.052).

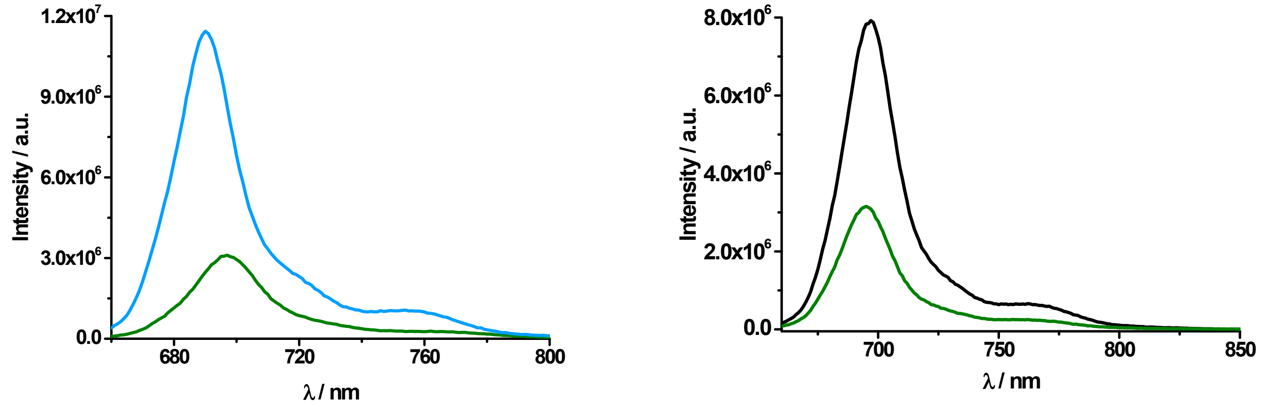

Fig. S5 Left: emission spectra of ZnP-ZnPc-$[Cu(phen)_2]^+$-C_{60} **2** (olive) and Zn^tBu_4Pc (cyan) in PhCN upon excitation at 650 nm (OD = 0.09); Right: emission spectra of reference compounds 5 (olive) and 6 (black) in PhCN upon excitation at 650 nm (OD = 0.06);

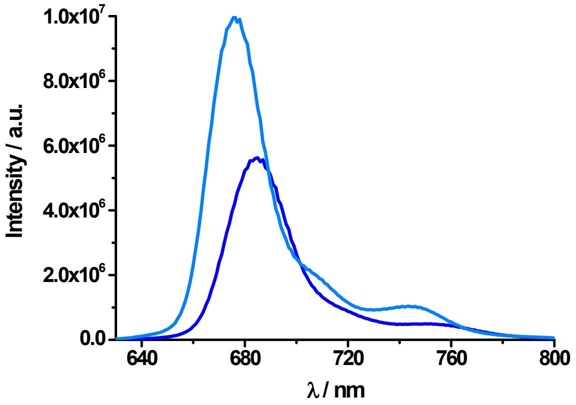

Fig. S6 Emission spectra of ZnPc-Fc-$[Cu(phen)_2]^+$-C_{60} **3** (blue) and the ZnPc reference **12** (cyan) in PhCN upon excitation at 610 nm (OD = 0.21).

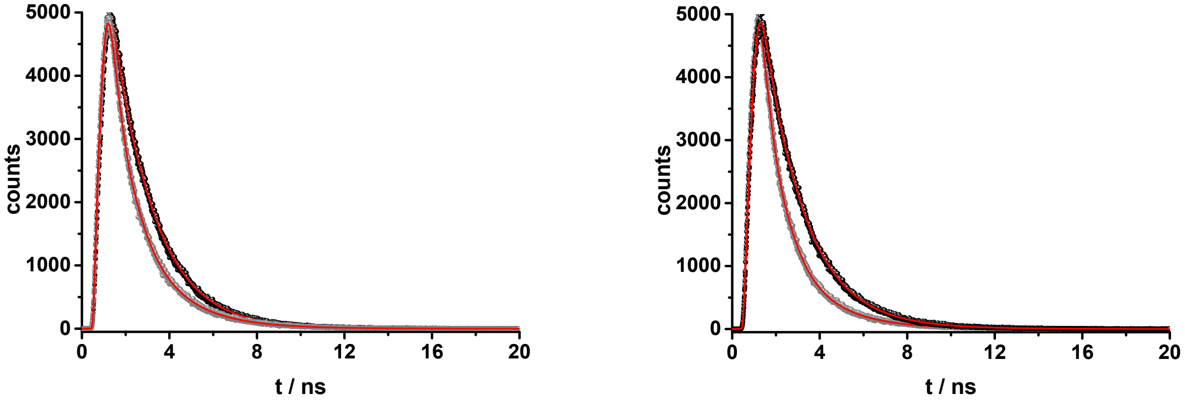

Fig. S7 Left: fluorescence decay of ZnTPP **11** (black) and Fc-ZnP-[Cu(phen)$_2$]$^+$-C$_{60}$ **1** (grey) and corresponding fits (red) upon excitation at 403 nm and detection at 600 nm in THF at RT. Right: fluorescence decay of reference compounds **5** (black) and **4** (grey) and corresponding fits (red) upon excitation at 403 nm and detection at 600 nm in THF at RT.

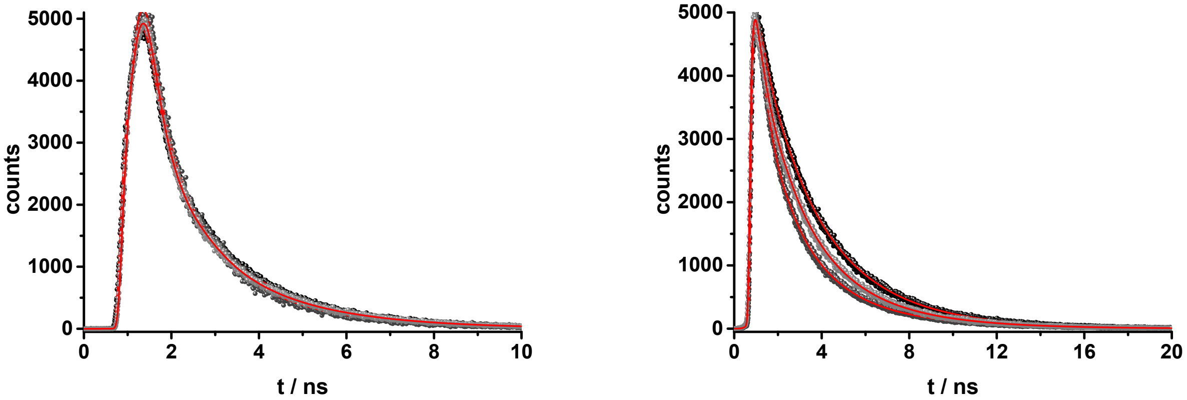

Fig. S8 Left: fluorescence decay of ZnP-ZnPc-[Cu(phen)$_2$]$^+$-C$_{60}$ **2** (grey), references **6** (dark grey) and **7** (grey)and corresponding fits (red) upon excitation at 403 nm and detection at 600 nm in THF at RT. Right: fluorescence decay of ZnP-ZnPc-[Cu(phen)$_2$]$^+$-C$_{60}$ **2** (grey), reference compounds **6** (dark grey) and **7** (grey) and corresponding fits (red) upon excitation at 647 nm and detection at 690 nm in THF at RT.

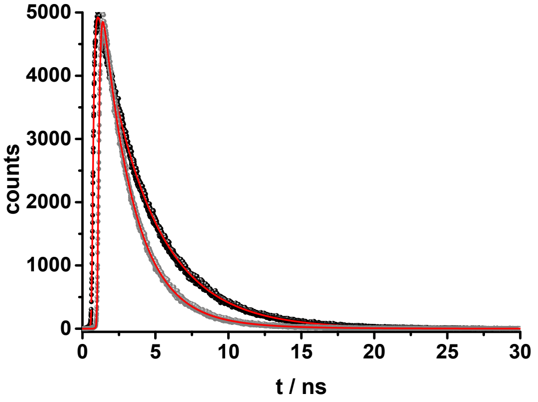

Fig. S9 Fluorescence decay of ZnPc **12** (black) and Fc-ZnPc-[Cu(phen)$_2$]$^+$-C$_{60}$ **3** (grey) and corresponding fits (red) upon excitation at 647 nm and detection at 680 nm in THF at RT.

4.2. Transient Absorption

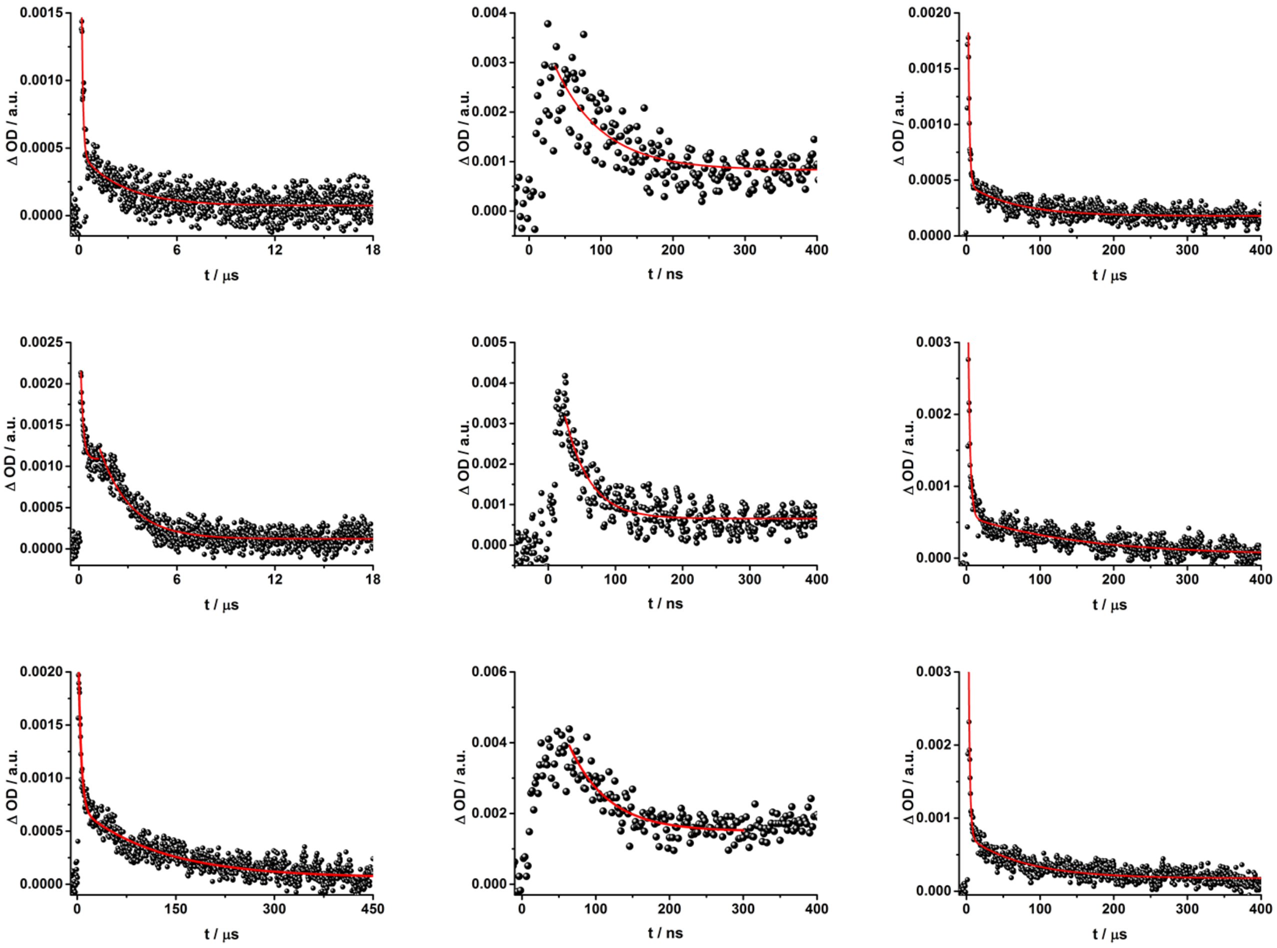

Fig. S10 Time absorption profiles of Fc-ZnP-$[Cu(phen)_2]^+$-C_{60} rotaxane 1 in tetrahydrofuran at room temperature under oxygen atmosphere (top), air (center) and argon atmosphere (bottom) at 680 nm (left) and 1010 nm (center and right), monitoring the charge recombination.

Table S3 Excited state lifetimes from ns transient absorption measurements on rotaxane **1** in THF under air, Ar and O_2 at room temperature.

	460 nm	680 nm		1010 nm		
O_2	111 ± 1 ns	110 ± 11 ns	2.5 ± 0.2 μs	67 ± 1 ns	2.1 ± 0.5 μs	60 ± 20 μs
Air	157 ± 19 ns	178 ± 21 ns	2.1 ± 0.3 μs	48 ± 3 ns	2.1 ± 0.1 μs	60 ± 12 μs
Ar	142 ± 12 μs	112 ± 14 μs	2.6 ± 0.1 μs	55 ± 3 ns	2.6 ± 0.1 μs	66 ± 8 μs
average	–	–	2.3 ± 0.3 μs	55 ± 8 ns	2.3 ± 0.4 μs	61 ± 16 μs
transient	**^{3}ZnP***	**^{3}ZnP***	**$ZnP^{•+}$**	**$C_{60}^{•-}$**	**$C_{60}^{•-}$**	**$C_{60}^{•-}$**

4.2.1. Reference compounds excited at 387 nm (fs-laser) and 355 nm (ns-laser)

The differential absorption spectra of C_{60} **10** are known from the literature.[3,5] Upon, for example, 387 nm fs-excitation the singlet excited state is formed immediately after the laser pulse with maxima at 510 and 920 nm. Within 1.7 ns the singlet excited state undergoes quantitative intersystem crossing to the energetically lower lying triplet excited state with a characteristic maximum at 720 nm. A triplet lifetime of 23 µs was determined upon 355 nm ns-excitation in argon saturated THF.

The differential absorption changes recorded upon 387 nm excitation of catenane **9** are dominated by characteristic MLCT transient absorptions. The latter include a broad maximum between 540 and 610 nm followed by another maximum around 910 nm. As a complement, transient bleaching at 440 and 700 nm corresponds to the MLCT absorption.[1, 8-11, 19] From the transient absorption changes a lifetime of 645 ns is derived upon ns-excitation and is attributed to the MLCT triplet excited state,[1] since it is well established that the MLCT singlet excited state transforms within hundreds of femtoseconds to the energetically lower lying MLCT triplet excited state.[20, 21]

Upon fs-excitation at 387 nm, the differential absorption spectra of **8** in THF are a combination of the transient changes recorded for references **9** and **10**. The transient observed at 590 nm is attributed to the ^{3*}MLCT, while the maxima at 530 and 920 nm correlate to the C_{60} singlet excited state. The latter decays rapidly with a lifetime of 40 ps due to the presence of $[Cu(phen)_2]^+$. The MLCT triplet excited state does not decay completely within the 7.5 ns time scale of our experimental setup. The peak at 1035 nm is the fingerprint absorption of the one-electron reduced C_{60}.[22] Thus, it is concluded that the deactivation of the C_{60} singlet excited state involves an electron transfer. The one-electron reduced form of C_{60} is stable on the time scale of the femtosecond experiments and decays in complementary ns-excitation experiments with a lifetime of 100 ns in THF.

For ZnPc **12** differential absorption changes evolve immediately after the 387 nm laser pulse, characterized by transient bleaching at 610 and 680 nm and an absorption maximum at 850 nm in THF, corresponding to the singlet excited state.[23] The latter decays within ~ 3 ns to populate the energetically lower-lying triplet excited state. The newly developing band at 480 nm reflects the diagnostic signature of the ZnPc triplet excited state with a lifetime of 120 µs.[23, 24]

4.2.2. Reference compounds excited at 420 nm (fs-laser)

ZnTPP **11** was excited with 420 nm fs-laser pulses. In this particular case, transient characteristics, which are formed immediately, include minima at 420, 560, and 600 nm, a maximum at 460 nm, and a broad absorption from 570 to 750 nm in THF. These correspond to the singlet excited state of ZnP.[1] Within 2 ns the ZnP singlet

excited state decays to the energetically lower lying ZnP triplet state with a 840 nm maximum and a 45 μs lifetime.[25,1]

When exciting **5** into the ZnP Soret band at 420 nm the visible part of the spectrum is dominated by the ZnP singlet excited state with maxima at 455, 580, and 635 nm and ground state bleaching with minima at 425, 560, and 600 nm. Here, the ZnP singlet excited state undergoes intersystem crossing within 1.7 ns to yield maxima at 470 and 850 nm. The lifetime of the latter exceeds the 7.5 ns time scale of the experimental setup.

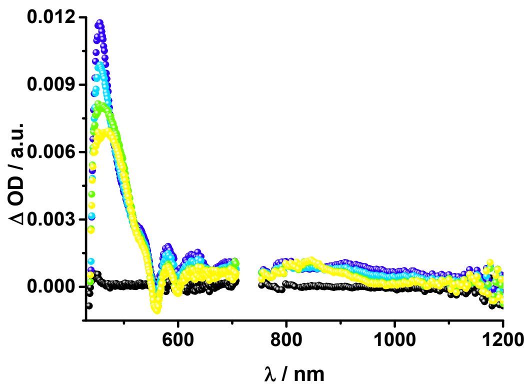

Fig. S11 Transient absorption spectrum (visible and near-infrared) of ZnP-Fc-(phen) reference **5** registered upon femtosecond flash photolysis (420 nm; 150 nJ) in THF with time delays between 0 (black) and 7.5 (yellow) ns at room temperature.

4.2.3. Reference compounds excited at 387 / 420 nm (fs-laser) and 425 nm (ns-laser)

Rotaxane **4** was excited at either 420 or at 387 nm. With the 420 nm excitation wavelength ZnP is mainly excited, while at 387 nm ZnP and $[Cu(phen)_2]^+$ both absorb. Still, excitation at either wavelength results in spectra, which are dominated by the characteristic ZnP signatures, as seen for model **5**. The ZnP singlet excited state decays via ISC within ~ 800 ps to give the triplet excited state of ZnP with its 460 and 850 nm absorption characteristics is discernable (Figure 6, right). Upon comparison with **5**, the rather broad transient in the near infrared is assigned to the MLCT triplet excited state. Considering that in **4** the ZnP singlet excited state lifetime is notably reduced relative to **5** we conclude that the presence of $[Cu(phen)_2]^+$ induces ZnP fluorescence quenching via energy transfer. However, no evidence was found in support of an electron transfer process. Thus, we conclude that energy transfer to $[Cu(phen)_2]^+$ dominates the excited state deactivation of ZnP in rotaxane **4**.

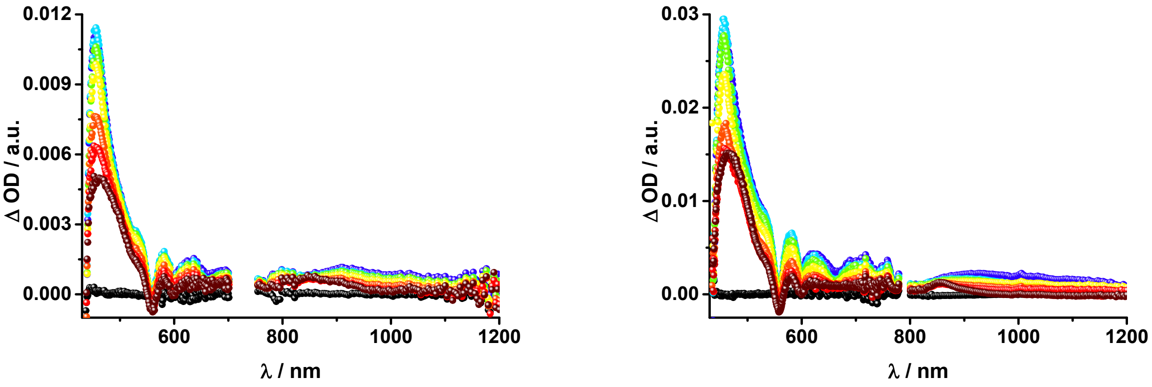

Fig. S12 Left: Transient absorption spectrum (visible and near-infrared) registered upon femtosecond flash photolysis (420 nm, 150 nJ) of ZnP-Fc-[Cu(phen)$_2$] rotaxane **3** in THF with time delays between 0 (black) and 7500 (wine) ps at room temperature. Right: Transient absorption spectrum (visible and near-infrared) registered upon femtosecond flash photolysis (387 nm, 200 nJ) of ZnP-Fc-[Cu(phen)$_2$] rotaxane **3** in THF with time delays between 0 (black) and 7500 (wine) ps at room temperature.

4.2.4. References excited at 387 / 420 / 660 nm (fs-laser) and 425 / 670 nm (ns-laser)

Upon exclusive excitation of ZnPc of ZnPc-phen-ZnP thread **7** at 660 nm the differential absorption spectra feature only ZnPc transients, namely its singlet excited state at ~ 800 nm with a lifetime 2.9 ns in THF and its triplet excited state at 490 nm including ground state bleaching at 680 nm.[24, 26-31] In contrast, upon excitation of **7** at 420 nm, which matches the ZnP Soret band, the visible part of the spectrum is dominated by the ZnP signature absorptions. The 450 nm ZnP singlet excited state undergoes energy transfer within 50 ps to yield the ZnPc singlet excited state with a comparably weak maximum at 840 nm and a lifetime of 3.1 ns in THF. This observation is in line with what was seen in the steady state emission experiments (*vide supra*). The ZnPc ground state bleaching at 610 and 680 nm develops within the same time span of 50 ps. Competitive with the energy transfer process the singlet excited state of ZnP decays via ISC resulting in the ZnP triplet excited state (~ 480 and 840 nm), which is stable over the window of the fs experimental setup (7.5 ns). Upon laser irradiation of **7** at 387 nm, all moieties are excited at the same time. Thus, a mixture of ZnP and ZnPc features is observed in the differential absorption spectra. To be more precise, maxima at 460, 580, and 630 nm correspond to the ZnP singlet excited state and decay within 50 ps. The ZnPc singlet excited state undergoes ISC to give the triplet manifold with its 500 and 840 nm absorption features within 1.7 ns. Transient bleaching is observed at 420, 560, and 610 nm as well as at 680 nm.

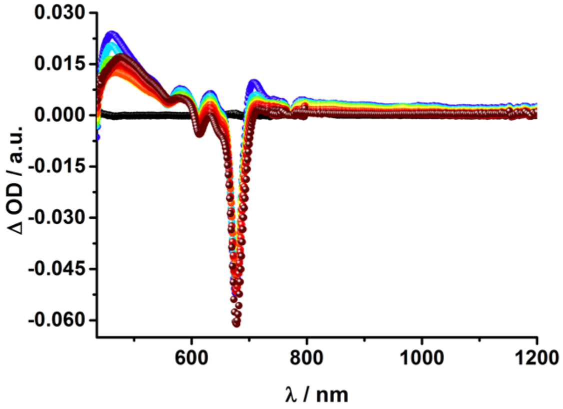

Fig. S13 Transient absorption spectrum (visible and near-infrared) registered upon femtosecond flash photolysis (387 nm, 200 nJ) of ZnP-ZnPc-(phen) thread **7** in tetrahydrofuran with time delays between 0 (black) and 7.5 ns (wine) at room temperature.

Excitation of rotaxane **6** at 420 nm yields the characteristic signatures of the ZnP singlet excited state at 450 nm that decay through energy transfer to give the rather weak singlet excited state of ZnPc with a lifetime of 1.0 ns, which is again in good agreement with the steady state experiments. In contrast to **7**, no triplet excited state features of ZnP could be observed. Instead the system decays exclusively via the ZnPc triplet excited state with its 480 nm fingerprint, which is stable on the 7.5 ns time scale. Additionally bleaching of ZnPc transients is observed at 610 and 680 nm. Upon exclusive excitation of ZnPc, in rotaxane **6** at 660 nm, the visible region is dominated by a broad peak maximizing at ~ 490 nm, which is stable over the time span of 7.5 ns and is assigned to the ZnPc triplet excited state. The ZnPc singlet excited state at 820 nm deactivates via ISC with 1.5 ns. At 680 nm, the ZnPc ground state bleaching is observed. At 387 nm all photoactive building blocks in rotaxane **6** were excited. Thus, the transient absorption spectra reveal a mixture of ZnP, ZnPc as well as $[Cu(phen)_2]^+$ features. As previously observed, the ZnP singlet excited state rapidly decays with 120 ps to give the ZnPc singlet excited state with a lifetime of 1.2 ns. The latter then decays via ISC to give the ZnPc triplet excited state, which is stable over the time scale of our fs setup. The MLCT absorption from $[Cu(phen)_2]^+$ exhibits a maximum at 910 nm, while in the visible part of the spectra it is masked by the ZnP and ZnPc transients. Since in **6** the ZnPc singlet state is significantly shorter lived than that seen for **7**, in line with time resolved fluorescence experiments, we conclude that the $[Cu(phen)_2]^+$ quenches the ZnPc fluorescence. No spectroscopic proof for charge separation is evident in the transient absorption spectra. Thus, only energy transfer to $[Cu(phen)_2]^+$ takes place in rotaxane **6**.

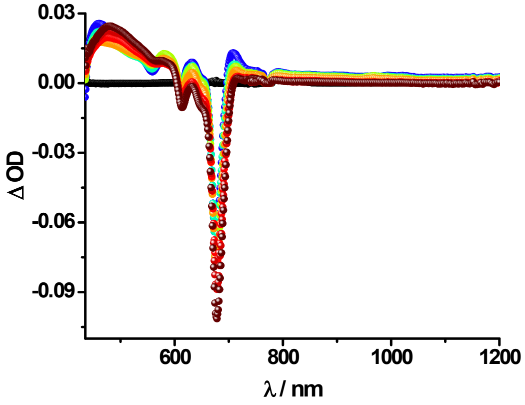

Fig. S 14 Transient absorption spectrum (visible and near-infrared) registered upon femtosecond flash photolysis (387 nm, 200 nJ) of ZnP-ZnPc-[Cu(phen)$_2$] rotaxane 6 in THF with time delays between 0 (black) and 7500 (wine) ps at room temperature.

5. Experimental Section

5.1. General Information and Materials

NMR spectra were obtained on either a Bruker AVANCE 400 (400 MHz) or an AVANCE 800 (800 MHz) spectrometer using deuterated solvents as the lock. The spectra were collected at 25°C unless otherwise noted and chemical shifts (δ, ppm) were referenced to residual solvent peak (^{1}H, $CDCl_3$ at 7.26 ppm; ^{13}C at 77.2 ppm). In the assignments, the chemical shift (in ppm) is given first, followed, in brackets, by multiplicity (s, singlet; d, doublet; t, triplet; q, quadruplet, m, multiplet; br, broad), the value of the coupling constants in Hz if applicable, the number of protons implied and finally the assignment. In the ^{1}H NMR assignment (δ), H_o and H_m refer to the hydrogen atoms at the *ortho* and *meta* positions, respectively, of the phenyl ring attached to the phenanthroline ring system, whose hydrogen atoms are numbered $H_{3,8}$, $H_{4,7}$, $H_{5,6}$, respectively. Ar is used as an abbreviation for aromatic ring. Mass spectra were obtained on an Agilent 1100 Series Capillary LCMSD Trap XCT Spectrometer in positive or negative-ion mode and ThermoFinnigan PolarisQ ion-trap GCMS Spectrometer. MALDI-TOF mass spectra were recorded in a Bruker OmniFLEX MALDI-TOF MS Spectrometer. This instrument was operated at an accelerating potential of 20 kV in linear mode. The mass spectra represent an average over 128 consecutive laser shots. Mentioned *m/z* values correspond to monoisotopic masses. The compound solutions (10^{-3} mol/L) were prepared in THF. Matrix compound was purchased from Aldrich and used without further purification. The matrix, α-cyano-4-hydroxy-cinnamic acid (CCA), was dissolved (10 g/L) in a solvent mixture composed of H_2O/CH_3CN/TFA (25/75/1, v/v). Two microliters of compound solution was mixed with 10 µL of matrix solution. The final solution was deposited onto the sample target and allowed to dry in air. All chemicals were purchased from Sigma-Aldrich, Alfa Aesar and Acros Organics and used without further purification. For moisture sensitive reactions, solvents were freshly distilled. Methylene chloride (CH_2Cl_2) and acetonitrile (CH_3CN) were dried over calcium hydride while tetrahydrofuran (THF) was dried using sodium/benzophenone. Anhydrous dimethylformamide (DMF) was used as received. All syntheses were carried out using Schlenk techniques. Moisture sensitive liquids were transferred by cannula or syringe. The progress of the reactions was monitored by thin-layer chromatography (TLC) whenever possible. TLC was performed using precoated glass plates (Silica gel 60, 0.25 mm thickness) containing a 254 nm fluorescent indicator. Column chromatography was carried out using Merck Silica gel 60 (0.063-0.200 mm). Catenane model compounds 8 and 9 were prepared following the conditions reported in our previous work.[31] Compounds **10**[17], **11**[32], **12**[33], **13**,[1, 34,] **16**[8, 9, 32] **18**[1], **20**[33] and **22**[10] were synthesized following literature procedures.

5.2. Synthesis

Compound 14. In a three-necked round bottom flask, compound **13** (1.00 g, 1.60 mmol), and triethylamine (2 mL) were dissolved in 60 mL of dry CH_2Cl_2 under N_2 atmosphere with magnetic stirring. In an addition funnel, *p*-toluenesulfonyl chloride (*p*-TsCl) (0.304 g, 1.60 mmol) was dissolved in 20 mL of dry CH_2Cl_2. The round bottom flask was cooled to 0°C and the *p*-TsCl solution was added dropwise (over 30 min) under N_2 atmosphere with magnetic stirring. After addition, the reaction mixture was kept at 0°C for 4 h and then allowed to warm to rt and stirred for 12 h. The reaction was carefully quenched at 0 °C by addition of 10% aqueous HCl. The organic phase was decanted, washed with water (3 x 50 mL), dried over Na_2SO_4, filtered through paper and concentrated under reduced pressure. Final purification was achieved by column chromatography (SiO_2) using CH_2Cl_2/MeOH as the eluent (gradient from 0 to 4%, v/v) to afford 14 as an orange oil (0.56 g, 45% yield). ^1H NMR ($CDCl_3$), δ ppm: 8.40 (d, *J* = 9.0 Hz, 4H, H_o); 8.27 (d, *J* = 9.0 Hz, 2H, H_4 and H_7); 8.09 (d, *J* = 9.0 Hz, 2H, H_3 and H_8); 7.77 (d, *J* = 9.0 Hz, 2H, *ortho* protons at Ar-OTs); 7.74 (s, 2H, H_5 and H_6); 7.34 (d, *J* = 9.0 Hz, 2H, *meta* protons at Ar-OTs); 7.12 (d, *J* = 9.0 Hz, 4H, H_m); 4.25–3.50 (set of signals, 24H, O–CH_2–CH_2–O); 2.39 (s, 3H, CH_3-Ar-OTs group). LC-MSD: m/z found 783.45 $[M+H]^+$, calculated 782.28 for $C_{43}H_{46}N_2O_{10}S$.

Compound 15. *Caution: Organic azides have been reported in literature as potential explosives. The authors suggest the use of standard PVC blast shield while handling organic azides.* Compound **14** (0.50 g, 0.64 mmol) and NaN_3 (0.125 g, 1.92 mmol) were dissolved in 15 mL of anhydrous DMF in a 100 mL Schlenk flask and the reaction mixture was heated at 80 °C for 12 h. The DMF was evaporated under reduced pressure. CH_2Cl_2 (50 mL) and H_2O (20 ml) was added, the organic phase was separated, washed with water (3 x 20 mL), dried over Na_2SO_4, filtered through paper and concentrated under reduced pressure. Final purification was achieved by flash chromatography (SiO_2) using CH_2Cl_2/MeOH (99:1, v/v) as the eluent, affording 15 as a light yellow solid (0.355 g, 85% yield). IR (KBr) ν cm^{-1}: 2095 (N=N stretch).

Compound 17. Porphyrin carboxylic acid 16 (0.140 g, 0.132 mmol), **15** (0.086g, 0.132 mmol), EDC (0.051 g, 0.267 mmol), and DMAP (0.033g, 0.270 mmol) were place in in a 100 mL Schlenk flask and 25 mL of dry CH_2Cl_2 was added under N_2 atmosphere. The purple mixture was stirred at room temperature for 12 hours. The solution was washed with aqueous 10% HCl, then with water (3 x 10 mL), dried over Na_2SO_4, filtered through paper and concentrated under reduced pressure. Final purification was achieved by chromatography (SiO_2) using a gradient of CH_2Cl_2/MeOH (99:1, v/v) as the eluent to afford 17 as a purple solid (0.145 g, 65% yield). ^1H NMR ($CDCl_3$), δ ppm: 8.94 (s, 4H, H-pyrrolic); 8.86 (d, *J* = 6.5 Hz, 2H, H-pyrrolic); 8.72 (d, *J* = 6.5 Hz, 2H, H-pyrrolic); 8.35 (d, *J* = 9.0 Hz, 2H, H-*ortho* at Ar-COO); 8.26 (d, *J* = 8.9 Hz, 4H, H_o); 8.19 (d, *J* = 8.9 Hz, 2H, $H_{4,7}$); 8.01 (s, 6H, H-*ortho* at Ar-*t*Bu); 7.82 (s, 2H, $H_{5,6}$); 7.71 (s, 3H, H-*para* at Ar-*t*Bu); 7.56 and 7.51(d, *J* = 8.9 Hz, 2H, $H_{3,8}$); 7.12 (d, *J* = 9.0 Hz, 2H, H-*meta* at Ar-COO); 7.03 (d, *J* = 8.9 Hz, 2H, H_m); 6.96 (d, *J* = 8.9 Hz, 2H, $H_{m'}$); 4.60-3.90 (set of signals totalizing 24H, O–CH_2–CH_2–O); 1.45 (s, 54H, *t*-Bu). MALDI-TOF: m/z found 1692.99 $[M+H]^+$, calculated 1692.14 for $C_{105}H_{113}N_9O_8Zn$. IR (KBr) ν cm^{-1}: 2091 (N=N stretch).

General Procedure for the Synthesis of Rotaxanes. In the reaction flask, the suitable phen-macrocycle derivative (1.0 equiv.) and $[Cu(CH_3CN)_4][PF_6]$ (1.0 equiv.) were dissolved in 1 mL of a mixture of CH_2Cl_2/CH_3CN (7:3, v/v) and the solution was stirred at room temperature under nitrogen atmosphere for 30 minutes. The appropriate phen-thread derivative (1.0 equiv.) was added as a solid and the reaction mixture was stirred at room temperature under nitrogen atmosphere for 12 h. TLC showed quantitative formation of the pseudo-rotaxane precursors. The solvents were evaporated under reduced pressure and the remaining solid was dissolved in 4 mL of CH_2Cl_2 under nitrogen atmosphere. The suitable alkynyl derivative (2.5 equiv.) was added to the reaction flask as a solid. Meanwhile, in a second flask, CuI (2.0 equiv.), sodium ascorbate (8.0 equiv.) and sulfonated bathophenanthroline (4.0 equiv.) were dissolved in 2 mL of degassed H_2O/ethanol mixture (1:1, v/v). The resulting pink suspension was heated at reflux for 2 min and cooled back to rt. The resulting dark red solution was transferred to the reaction flask by syringe along with 1,8-diazabicycloundec-7-ene (DBU) (2.0 equiv.). The reaction mixture was stirred at room temperature under nitrogen atmosphere for 12 h. The crude mixture was neutralized by adding 10% HCl aqueous solution and extracted with CH_2Cl_2 (3 x 15 mL). The organic phase was washed with water (3 x 15 mL), concentrated to a volume of 5 mL and then stirred for 3 h with a saturated MeOH solution of KPF_6 (10 mL) to effect the anion exchange. The solvents were evaporated under reduced pressure, the remaining insoluble solid was extracted with CH_2Cl_2 (3 x 50 mL) and filtered through paper. The solvent was evaporated under reduced pressure and the crude product was purified by column chromatography (SiO_2) using appropriate CH_2Cl_2/CH_3OH mixture for each case as eluent. The excess alkynyl derivative was eluted first, followed by the corresponding thread compound, whose isolation indicates that some unthreading occurred during rotaxane assembly. The target rotaxane was the third eluted product from the column.

Rotaxane 1. This compound was prepared from macrocycle **18** (0.015 g, 10.6 μmol), thread **17** (0.018 g, 10.6 μmol) and commercially available alkynylferrocene (0.006 g, 26.5 μmol) following the general procedure for rotaxanes synthesis. Final purification was achieved by column chromatography (SiO_2) using a gradient of CH_2Cl_2/CH_3OH (98:2, v/v) to yield the target compound as a dark red solid (0.034 g, 90% yield). MALDI-TOF: m/z found 3379.04 $[M – PF_6]^+$, calculated 3379.35 for $C_{216}H_{161}N_{11}O_{18}CuFeZn$.

Rotaxane 2. This compound was prepared from macrocycle **18** (0.015 g, 10.6 μmol), thread 17 (0.018 g, 10.6 μmol) and phthalocyanine **20** (0.020 g, 26.5 μmol) following the general procedure for rotaxanes synthesis. Final purification was achieved by column chromatography (SiO_2) using a gradient of CH_2Cl_2/CH_3OH (98:2, v/v) to yield the target compound as a deep blue solid (0.031 g, 72% yield). MALDI-TOF: m/z found 3939.90 $[M – PF_6]^+$, calculated 3939.25 for $C_{250}H_{191}N_{19}O_{18}CuZn_2$.

Rotaxane 3. This compound was prepared from macrocycle **18** (0.015 g, 10.6 μmol), thread 22 (0.009 g, 10.6 μmol) and phthalocyanine **20** (0.020 g, 26.5 μmol) following the general procedure for rotaxanes synthesis. Final purification was achieved by column chromatography (SiO_2) using a gradient of CH_2Cl_2/CH_3OH (97:3, v/v) to yield

the target compound as a green solid (0.028 g, 81% yield). MALDI-TOF: m/z found 3110.99 [M – PF_6]$^+$, calculated 3110.98 for $C_{192}H_{125}N_{15}O_{18}CuFeZn$.

Rotaxane 4. This compound was prepared from pentaethyleneglycol phen-macrocycle[35] (0.006 g, 10.6 µmol), thread **17** (0.018 g, 10.6 µmol) and commercially available alkynylferrocene (0.006 g, 26.5 µmol) following the general procedure for rotaxanes synthesis. Final purification was achieved by column chromatography (SiO_2) using a gradient of CH_2Cl_2/CH_3OH (98:2, v/v) to yield the target compound as a red solid (0.025 g, 88% yield). MALDI-TOF: m/z found 2532.22 [M – PF_6]$^+$, calculated 2531.72 for $C_{151}H_{157}N_{11}O_{14}CuFeZn$.

Rotaxane 6. This compound was prepared from pentaethyleneglycol phen-macrocycle[35] (0.006 g, 10.6 µmol), thread **17** (0.018 g, 10.6 µmol) and phthalocyanine 20 (0.020 g, 26.5 µmol) following the general procedure for rotaxanes synthesis. Final purification was achieved by column chromatography (SiO_2) using a gradient of CH_2Cl_2/CH_3OH (97:3, v/v) to yield the target compound as a blue solid (0.025 g, 88% yield). MALDI-TOF: m/z found 3091.82 [M – PF_6]$^+$, calculated 3091.24 for $C_{185}H_{187}N_{19}O_{14}CuZn_2$.

Compound 5. This thread model compound was isolated (red solid) as a byproduct from the synthesis of rotaxane **4** (0.002 g, 9% yield). To remove any copper contamination from the phen coordinating sites, the isolated material was dissolved in 2 mL of CH_2Cl_2/CH_3CN (1:1, v/v) mixture at room temperature and a saturated KCN aqueous solution (2 mL) was added. The resulting mixture was stirred at room temperature for 12 h. About 10 mL of CH_2Cl_2 was added to the crude mixture, the organic layer was decanted, copiously washed with water (5 x 10 mL), dried over Na_2SO_4, filtered through paper and concentrated under reduced pressure. Final purification was achieved by flash chromatography (SiO_2) using a gradient of CH_2Cl_2/MeOH (99:1, v/v) as the eluent to afford quantitatively the target compound as a red solid. Inductively-coupled plasma mass spectrometry (ICP-MS) analysis revealed a copper residual level of less than 250 ppm, which was a level acceptable for the spectroscopic investigation. MALDI-TOF: m/z found 1903.44 [M + H]$^+$, calculated 1902.22 for $C_{117}H_{123}N_9O_8FeZn$.

Compound 7. This thread model compound was isolated (blue solid) as a byproduct from the synthesis of rotaxane **6** (0.006 g, 22% yield). To remove any copper contamination from the phen coordinating sites, the isolated material was dissolved in 4 mL of CH_2Cl_2/CH_3CN (7:3, v/v) mixture at room temperature and a saturated KCN aqueous solution (4 mL) was added. The resulting mixture was stirred at room temperature for 12 h. About 10 mL of CH_2Cl_2 was added to the crude mixture, the organic layer was decanted, copiously washed with water (5 x 10 mL), dried over Na_2SO_4, filtered through paper and concentrated under reduced pressure. Final purification was achieved by flash chromatography (SiO_2) using a gradient of CH_2Cl_2/MeOH (99:1, v/v) as the eluent to afford quantitatively the target compound as a blue solid. Inductively-coupled plasma mass spectrometry (ICP-MS) analysis revealed a copper residual level of less than 240 ppm, which was a level acceptable for the spectroscopic investigation. MALDI-TOF: m/z found 2479.95 [M + H_2O]$^+$, calculated 2461.74 for $C_{151}H_{153}N_{17}O_8Zn_2$.

5.3. Electrochemical and Photophysical Studies

All solvents used were purchased from commercial suppliers (spectroscopic grade; 99.5 %) and used without further purification. A single-compartment, three electrode cell configuration was used for the square wave voltammetry measurements, using a glassy carbon electrode (3 mm diameter) as a working electrode, a platinum wire as a counter and a silver wire as a reference electrode. All electrochemical measurements were performed with a METROHM FRA 2 µAutolab Type III potentiostat. For the photophysical characterization the samples were placed in fluorimetric cuvettes with different pathways and, when necessary, purged with molecular oxygen or argon. Steady-state UV-vis absorption spectroscopy was performed on a Lambda2 spectrometer (Perkin Elmer). Steady state fluorescence spectra were carried out at a FluoroMax3 spectrometer (Horiba) in the visible detection range and at a FluoroLog3 spectrometer (Horiba). Fluorescence lifetimes were determined by the time correlated single photon counting technique using a FlouroLog3 emission spectrometer (Horiba JobinYvon) equipped with an R3809U-58 MCP (Hamamatsu) and an N-405LH laser diode (Horiba JobinYvon) exciting at 403 nm (300 ps fwhm) or a SuperK Extreme high power supercontinuum fiber laser EXW-12 (NKT) exciting at 420 nm (150 ps fwhm). Femtosecond transient absorption (TA) experiments were carried out with an amplified Ti:Sapphire laser system CPA-2110 fs laser (Clark MXR: output 775 nm, 1 kHz, and 150 fs pulse width) using a transient absorption pump/probe detection system (TAPPS Helios, Ultrafast Systems). The 420 nm excitation wavelength was generated with a noncolinear optical parametric amplifier (NOPA, Clark MXR). For the, excitation wavelength the energy of 150-200 nJ/ pulse was selected. Nanosecond transient absorption laser photolysis measurements were performed with the output from an optical parametric oscillator (OPO, Rainbow VIR, Opotek/Quantel, output: 425 and 670 nm, 5 mJ/pulse) pumped by the third harmonic (355 nm) of a Nd/YAG laser (Brilliant, Quantel). The optical detection was based on a pulsed (pulser MSP 05, Müller Elektronik-Optik) xenon lamp (XBO 450, Osram), a monochromator (Spectra Pro 2300i, Acton Research), a R928 photomultiplier tube (Hamamatsu Photonics), or a fast InGaAs photodiode (Nano 5, Coherent) with 300 MHz amplification, and a 1 GHz digital oscilloscope (WavePro7100, LeCroy). The experiments were performed on solutions in a 5x10 mm quartz glass cuvette.

6. Literature

1 J. D. Megiatto, Jr. D. I. Schuster, S. Abwandner, G. de Miguel and D. M. Guldi, *J. Am. Chem. Soc.*, 2010, **132**, 3847-3861.

2 J. D. Megiatto, Jr., R. Spencer and D. I. Schuster, *Org. Lett.*, 2009, **11**, 4152-4155.

3 J. D. Megiatto, Jr. and D. I. Schuster, *Chemistry*, 2009, **15**, 5444-5448.

4 F. Bitsch, C. O. Dietrich-Buchecker, A. K. Khemiss, J.-P. Sauvage and A. Van Dorsselaer, *J. Am. Chem. Soc.*, 1991, **113**, 4023-4025.

5 J. D. Megiatto and D. I. Schuster, *New J. Chem.*, 2010, **34**, 276.

6 E. J. Yoo, M. Ahlquist, S. H. Kim, I. Bae, V. V. Fokin, K. B. Sharpless and S. Chang, *Angew. Chem. Int. Ed. Engl.*, 2007, **46**, 1730-1733.

7 L. Flamigni, A. M. Talarico, J. C. Chambron, V. Heitz, M. Linke, N. Fujita and J.-P. Sauvage, *Chemistry*, 2004, **10**, 2689-2699.

8 K. Li, P. J. Bracher, D. M. Guldi, M. A. Herranz, L. Echegoyen and D. I. Schuster, *J. Am. Chem. Soc.*, 2004, **126**, 9156-9157.

9 K. Li, D. I. Schuster, D. M. Guldi, M. A. Herranz and L. Echegoyen, *J. Am. Chem. Soc.*, 2004, **126**, 3388-3389.

10 J. D. Megiatto, Jr., K. Li, D. I. Schuster, A. Palkar, M. A. Herranz, L. Echegoyen, S. Abwandner, G. Miguel and D. M. Guldi, *J. Phys. Chem. B*, 2010, **114**, 14408-14419.

11 J. D. Megiatto, Jr., D. I. Schuster, G. d. Miguel, S. Wolfrum and D. M. Guldi, *Chem. Mater.*, 2012, **24**, 2472-2485.

12 D. I. Schuster, K. Li, D. M. Guldi and J. Ramey, *Org. Lett.*, 2004, **6**, 1919-1922.

13 J. D. Megiatto Jr., R. Spencer and D. I. Schuster, *J. Mater. Chem.*, 2011, **21**, 1544.

14 C. Ornelas, J. Ruiz Aranzaes, E. Cloutet, S. Alves and D. Astruc, *Angew. Chem. Int. Ed. Engl.*, 2007, **46**, 872-877.

15 C. O. Dietrich-Buchecker and J.-P. Sauvage, *Chem. Rev.*, 1987, **87**, 795-810.

16 L. Echegoyen and L. E. Echegoyen, *Acc. Chem. Res.*, 1998, **31**, 593-601.

17 A. Hirsch and M. Brettreich, *Fullerenes: Chemistry and Reactions*, Wiley-VCH, Weinheim, 2005

18 S. Kirner, M. Sekita and D. M. Guldi, *Adv. Mater.*, 2014, **26**, 1482-1493.

19 S. V. Kirner, D. M. Guldi, J. D. Megiatto, Jr. and D. I. Schuster, *Nanoscale*, 2015, **7**, 1145-1160.

20 N. Armaroli, M. A. J. Rodgers, P. Ceroni, V. Balzani, C. O. Dietrich-Buchecker, J.-M. Kern, A. Bailal and J.-P. Sauvage, *Chem. Phys. Lett.*, 1995, **241**, 555-558.

21 T. Gunaratne, M. A. J. Rodgers, D. Felder, J.-F. Nierengarten, G. Accorsi and N. Armaroli, *Chem. Commun.*, 2003, 3010.

22 T. Kato, T. Kodama, T. Shida, T. Nakagawa, Y. Matsui, S. Suzuki, H. Shiromaru, K. Yamauchi and Y. Achiba, *Chem. Phys. Lett.*, 1991, **180**, 446-450.

23 Y. Rio, W. Seitz, A. Gouloumis, P. Vazquez, J. L. Sessler, D. M. Guldi and T. Torres, *Chemistry*, 2010, **16**, 1929-1940.

24 A. M. V. M. Pereira, A. R. M. Soares, A. Hausmann, M. G. P. M. S. Neves, A. C. Tomé, A. M. S. Silva, J. A. S. Cavaleiro, D. M. Guldi and T. Torres, *Phys. Chem. Chem. Phys.*, 2011, **13**, 11858.

25 C. Luo, D. M. Guldi, H. Imahori, K. Tamaki and Y. Sakata, *J. Am. Chem. Soc.*, 2000, **122**, 6535-6551.

26 A. Hausmann, A. R. Soares, M. V. Martinez-Diaz, M. G. Neves, A. C. Tome, J. A. Cavaleiro, T. Torres and D. M. Guldi, *Photochem Photobiol Sci*, 2010, **9**, 1027-1032.

27 M. Quintiliani, A. Kahnt, T. Wolfle, W. Hieringer, P. Vazquez, A. Gorling, D. M. Guldi and T. Torres, *Chemistry*, 2008, **14**, 3765-3775.

28 W. Seitz, A. Kahnt, D. M. Guldi and T. Torres, *J. Porphyrins Phthalocyanines*, 2009, **13**, 1034-1039.

29 R. F. Enes, J. J. Cid, A. Hausmann, O. Trukhina, A. Gouloumis, P. Vazquez, J. A. Cavaleiro, A. C. Tome, D. M. Guldi and T. Torres, *Chemistry*, 2012, **18**, 1727-1736.

30 A. J. Jimenez, M. L. Marcos, A. Hausmann, M. S. Rodriguez-Morgade, D. M. Guldi and T. Torres, *Chemistry*, 2011, **17**, 14139-14146.

31 A. M. Pereira, A. Hausmann, A. R. Soares, J. P. Tome, O. Trukhina, M. Urbani, M. G. Neves, J. A. Cavaleiro, D. M. Guldi and T. Torres, *Chemistry*, 2012, **18**, 3210-3219.

32 J. S. Lindsey, *Acc. Chem. Res.*, 2010, **43**, 300-311.

33 E. M. Maya, P. Vázquez and T. Torres, *Chem. Eur. J.*, 1999, **5**, 2004-2013.

34 C. O. Dietrich-Buchecker and J.-P. Sauvage, *Tetrahedron*, 1990, **46**, 503-512.

35 C. O. Dietrich-Buchecker and J.-P. Sauvage, *J. Am. Chem. Soc.*, 1984, **106**, 3043-3045.
